# One-Step Formation of 2D/3D Perovskite Heterojunction via Ligand Intercalation and Facet Engineering for Efficient Perovskite Solar Cells

**DOI:** 10.1007/s40820-025-02058-8

**Published:** 2026-02-09

**Authors:** Drajad Satrio Utomo, Yanping Liu, Andi Muhammad Risqi, Mohammed Ghadiyali, Imil Fadli Imran, Rakesh Rosan Pradhan, Shynggys Zhumagali, Sofiia Kosar, Vladyslav Hnapovskyi, Christopher E. Petoukhoff, Hao Tian, Xiaoming Chang, Badri Vishal, Adi Prasetio, Anil Reddy Pininti, Marco Marengo, Ahmed Ali Said, Aleksandra Oranskaia, Jongbeom Kim, Chuanxiao Xiao, Frédéric Laquai, Thomas D. Anthopoulos, Udo Schwingenschlögl, Sang Il Seok, Randi Azmi, Stefaan De Wolf

**Affiliations:** 1 Physical Science and Engineering (PSE), King Abdullah University of Science and Technology (KAUST), Thuwal, 23955-6900 Kingdom of Saudi Arabia; 2 Center for Renewable Energy and Storage Technologies (CREST), King Abdullah University of Science and Technology (KAUST), 23955-6900 Thuwal, Saudi Arabia; 3 School of Science and Engineering, The Chinese University of Hong Kong (Shenzhen), Shenzhen, 518172 Guangdong People’s Republic of China; 4 School of Energy and Chemical Engineering, Ulsan National Institute of Science and Technology (UNIST), 50 UNIST-Gil, Eonyang-Eup, Ulju-Gun, Ulsan, 44919 Republic of Korea; 5 Ningbo Institute of Materials Technology and Engineering, Chinese Academy of Sciences, Ningbo, 315201 People’s Republic of China; 6 Ningbo New Materials Testing and Evaluation Center Co. Ltd, Ningbo, 315201 People’s Republic of China; 7 Physical Chemistry and Spectroscopy of Energy Materials, Ludwig Maximilians University Munich, Butenandtstraße 11 (E), 81377 Munich, Germany; 8 Department of Electrical and Electronic Engineering, Henry Royce Institute, Photon Science Institute, The University of Manchester, Manchester, M13 9PL UK

**Keywords:** Perovskite solar cells, 2D/3D perovskite heterojunction, 2D passivation, Low-dimensional perovskite, Perovskite crystallization

## Abstract

Two-dimensional/three-dimensional (2D/3D) perovskite heterojunctions at the contact interfaces have been proven to enhance the stability and power conversion efficiency (PCE) of perovskite solar cells (PSCs). The 2D/3D bilayer is typically formed via a solution post-treatment onto the 3D perovskite, where the 2D layer’s dimensionality depends on the ligand size and its reactivity. Despite their stability, long-chain ligands typically form 2D perovskites with low dimensionality (*n* = 1, 2) which feature poor charge conductivity and mobility. Here, we propose an in situ fabrication method incorporating long-chain oleylammonium (OlyA^+^) ligands directly into the perovskite ink. This approach forms 2D perovskite with higher dimensionalities (*n* ≥ 3) with enhanced (001) crystal facet orientation of the 3D film, improved energetic alignment, charge extraction, and structural stability. The fabricated inverted PSCs with 1.55 eV bandgap achieved a maximum PCE of 26.22% for small area and 24.6% for 1cm^2^ devices, as well as 21.1% for mini-modules (6.8 cm^2^). Additionally, the PSCs with in situ formed 2D/3D perovskite heterojunctions retained 90% and 80% of their initial PCE after 1200 h photothermal stability and 1050 h outdoor testing, respectively. Our one-step strategy produces uniform and stable 2D/3D perovskite heterojunctions with enhanced passivation capability, overcoming the limitations of conventional sequential methods and offering a promising and effective approach for highly stable and efficient PSCs.

**Supplementary Information:**

The online version contains supplementary material available at 10.1007/s40820-025-02058-8.

## Highlights

- One-step in-situ incorporation of long-chain oleylammonium ligands enables simultaneous crystallographic orientation control and 2D/3D heterojunction formation in inverted perovskite solar cells.
- Facet engineering promotes dominant (001) orientation and higher-dimensional 2D phases (n ≥ 3) for improved energy alignment and charge extraction.
- Achieved power conversion efficiencies of 26.22% (0.1 cm^2^), 24.6% (1 cm^2^), and 21.1% (6.8 cm^2^ mini-modules) with excellent photothermal and outdoor stability.

**Supplementary Information:**

The online version contains supplementary material available at 10.1007/s40820-025-02058-8.

## Graphical abstract

**Figure:**
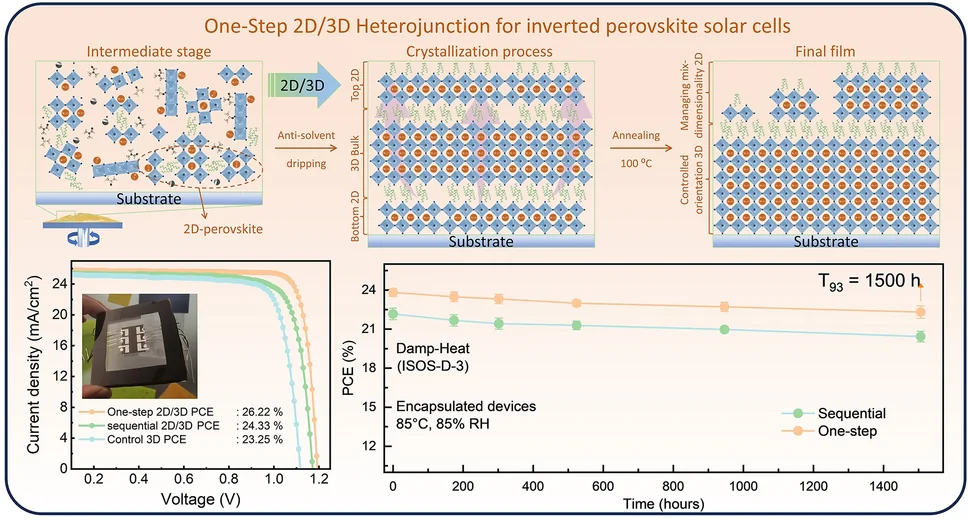

## Introduction

Inverted perovskite solar cells (PSCs) have attracted significant attention from both industry and academia due to their scalability, low-temperature processing, and their role in achieving many of the highest reported power conversion efficiencies (PCEs) for perovskite single-junction and tandem solar cells [1–4]. Many of the recent advances in PCEs and stability of PSCs have been driven by the development of two-dimensional/three-dimensional (2D/3D) perovskite heterojunctions, integrated at the top-contact interface [5–10]. The 2D/3D bilayer structure effectively mitigates the detrimental effects associated with surface and interface defects, suppressing nonradiative recombination, and improving the performance and stability at the device level. Conventionally, at the top surface, these bilayer configurations are developed via sequential solution post-treatment of the pre-formed 3D perovskite, involving bulky ammonium ligands [5, 6, 11–15]. However, this method faces several challenges, including excess ligand accumulation at the perovskite surface, poor crystallization control due to limited ligand penetration, and non-uniform coverage of the 2D capping layer due to surface termination and roughness of the 3D perovskite surface [5, 10, 12, 14, 16–20]. The large spatial variation of the 3D surface composition (i.e., excessive lead iodide, A-site terminated region, halide terminated region, etc.) can lead to uneven ligand adsorption and reaction, resulting in incomplete coverage and phase separation of 2D capping layers.

The sequential solution post-treatment of 2D/3D perovskite also strongly relies on the molecular size and reactivity of the ammonium ligands, which determines their penetration depth into 3D perovskite lattice and reaction to form a thin 2D capping layer [10, 12]. Short-chain ligands exhibit excessive penetration into the 3D perovskite, disrupting its lattice and compromising its structural integrity. This often necessitates the addition of an intermediate layer, such as cross-linkable polymers, to separate the 2D and 3D phases [8]. Conversely, long-chain ligands exhibit a slow diffusion rate and limited penetration due to steric hindrance, hence, they tend to remain near the surface and predominantly form 2D perovskites with lower-dimensionality (*n* = 1 or 2, where *n* represents the number of stacks of PbI_6_ slabs) [8, 21]. Although sequential 2D/3D structures improve the structural stability near the perovskite surface, they offer limited control over the crystallization of the 3D perovskite due to insufficient penetration depth, leaving the underlying bulk of the 3D perovskite untreated [22, 23]. This limited modulation hampers the overall film quality, resulting in heterogeneous crystal orientation [(001), (110), and (111)] and suboptimal optoelectronic properties [24–26]. Thus, it is critical to design a passivation technique that can direct the crystallographic orientation of the bulk of 3D perovskite.

Recent studies have demonstrated several strategies tailored to control the crystal orientation of perovskites, especially favoring vertical growth along the (001) crystallographic plane axis [24, 25, 27–30]. This orientation is particularly desirable for the device due to its superior charge transport properties and lower trap-state densities, resulting in more efficient carrier extraction compared to the other orientations like, (110) or (111) [25, 31]. The (001) facet offers a more continuous and less defective octahedral connectivity, whereas (110) and (111) planes introduce more distortions and grain boundaries that act as nonradiative recombination centers, thereby limiting the device performance. However, these compositional engineering strategies are often implemented separately from the surface post-treatment and integrating both functionalities typically add complexity to the crystallization dynamics and fabrication process. This challenge underscores the need for more streamlined approaches to achieve effective crystallographic orientation control and interface passivation.

Here, we introduce a spontaneous in situ formation of a 2D/3D bilayer at the top interface by incorporating oleylammonium (OlyA^+^) iodide as an aliphatic long-chain ligand into the perovskite ink, thereby eliminating post-deposition treatments and simplifying the fabrication process, while enabling better ligand distribution on the 3D perovskite surface. Due to its long alkyl chain and moderate reactivity, OlyA^+^ can modulate the 3D perovskite nucleation during early-stage crystallization while self-assembling at the top and near-the-top surface due to the lower interaction-energy with the PbI_2_. This promotes the spontaneous formation of a 2D/3D perovskite bilayer during annealing, which passivates the uppermost surface and simultaneously induces (001) crystal orientation in the underlying 3D perovskite. Unlike the sequential technique, the one-step method allows uniform ligand distribution and better integration into the crystallization process. The resulting 2D/3D perovskite bilayer has suppressed the formation of (111) crystal domains, with direct growth in the (001) plane. Additionally, the one-step 2D/3D process promotes the formation of a 2D perovskite with higher *n* value (predominantly *n* = 3), which can also improve charge extraction capabilities due to their deeper conduction band minimum (CBM). As a result, the inverted PSCs fabricated with a one-step 2D/3D strategy achieved a high PCE of 26.22% for 0.1 cm^2^, 24.6% for 1 cm^2^, and 21.1% for 6.8 cm^2^ mini-modules. Furthermore, encapsulated PSCs retained 90% of their initial PCE after 1200 h of photothermal degradation and 80% after 1050 h of outdoor exposure, indicating significantly improved operational stability. In contrast, PSCs prepared via the sequential post-treatment exhibited a lower initial PCE of 24.3% (0.1 cm^2^), with comparable stability. This highlights the advantage of the new approach in forming a more uniform and efficient 2D/3D perovskite bilayer with superior defect passivation, effectively overcoming the limitations of traditional sequential deposition techniques without sacrificing the stability.

## Experimental Section

### Materials

Lead iodide (PbI_2_, ultra-dry) was purchased from Alfa Aesar. Formamidinium iodide (FAI) and Methylammonium iodide (MAI) were purchased from Great Solar Ltd. Dimethylformamide (DMF, 99.8%, anhydrous), dimethylsulfoxide (DMSO, 99.9%, anhydrous), Trichloromethylene(TCM, 99.9%, anhydrous), and Cesium iodide (CsI, 99.9) were all purchased from Sigma-Aldrich. Oleylammonium Iodide (OlyAI, C_18_H_40_NI) and Methylammonium Chloride (MACl) were purchased from Xi’an Polymer L.T. 4PADCB ((4-(7H-dibenzo[c,g]carbazol-7-yl)butyl)phosphonic acid, > 98.0%) was purchased from Dyenamo. C_60_ (> 99.5% purity) and bathocuproine (BCP, > 99%) were purchased from Ossila Ltd.

### Device Fabrication

Glass/ITO substrates were washed following our standard cleaning process using soap, deionized (DI) water, acetone, and isopropyl alcohol for 15 min each, followed by nitrogen flow drying. Before fabrication, the substrate was exposed to UV-O for 10 min to remove some organic contaminants and improve adhesion. The 4PADCB (0.5 mg mL^−1^) was dissolved in ethanol solvent and shaken for 2 h before use. Then, the HTL SAMs were spin-coated onto the Glass/ITO at 5,000 rpm for 30 s in a controlled glove box, followed by thermal annealing at 100 °C for 10 min. After cooling down, the SAMs were washed with ethanol under dynamic coating at 4,000 rpm for 25 s to remove unbound SAMs. Then, the perovskite solution of Cs_0.025_MA_0.075_FA_0.90_PbI_3 +_ 0.4 mg mL^−1^ of OlyAI (1.5 M) and 20 mol% of MACL was prepared by dissolving them in a mixed solvent of DMF and DMSO (4:1 v/v ratio). The perovskite solution was shaken by a vortex shaker for 12 h at room temperature and in dark conditions. Then, the perovskite was spin-coated at 2,000 rpm for 40 s and 6,000 rpm for 10 s. The 200 mL of anisole was dynamically dropped onto the film during the transition to 6,000 rpm. Next, the perovskite film is annealed at 120 °C for 30 min to form a perovskite layer. As a comparison, the traditional sequential 2D/3D post-treatment was fabricated by coating OlyA^+^ ligand (0.5 mg mL^−1^ in TCM) onto Cs_0.025_MA_0.075_FA_0.90_PbI_3_ dynamically without further post-annealing, following our previous publication [5]. The perovskite film was stored at the vacuum chamber (10^−6^ Torr) for 4 h to remove the remaining solvent. For electron selective contact, the sample was transferred into a thermal evaporator for C_60_ (25 nm) and BCP (5 nm) and followed by top Ag (120 nm). The 120 nm MgF_2_ was also evaporated from the glass side as the anti-reflectance film to maximize the light absorption.

### Device Analysis

The *J-V* curve was measured using a Keithley 2400 source unit equivalent to AM 1.5 G (100 mW cm^−2^) light intensity with artificial sunlight Abet Technologies Sun 3000. The light was then calibrated by using mono-silicon standard cells (Newport). All devices were measured under forward and reverse scan rates with 0.1 V s^−1^ and dwell of 0.01 V. The measurement was done using an aperture mask with an area 0.062 cm^2^ (which was measured using optical microscope). The stabilized power output (SPO) was determined by recording the power output of the illuminated device at a fixed voltage near the maximum power point (MPP) extracted from the *J-V* curve. The external quantum efficiency (EQE) spectra were obtained by a lab-design optical breadboard equipped with 400 W Xenon lamp through a monochromator and filter inside an N_2_-filled glove box, and the lamp was calibrated to 603,621 Silicon and Germanium reference detector. The carrier mobility and trap density were measured using a Keithley 2400 source unit under dark conditions with voltage swap from 0.001 to 10 V simultaneously. The space-charge limited current voltage measurement was performed under dark conditions involving hole-only (ITO/PTAA/Perovskite/Spiro-OMeTAD/Ag) and electron-only (ITO/SnO_2_/Perovskite/C60/Ag) devices. The dark current–voltage sweep performed from 0.001 to 10 V.

Cross-sectional KPFM was performed using a lab-made setup integrated with a Bruker Dimension Icon atomic force microscope. All sample handling and measurement took place in an inert glovebox. A Pt-Ir-coated silicon probe (PPP-EFM) was used in tapping mode. Scans were conducted with a resolution of 1,024 pixels along the fast-scan axis and more than 32 lines along the slow-scan axis, using a scan rate of 0.35 Hz. To achieve a clean cross-section that exposed all layers of the device, the sample was cleaved directly, without polishing or ion milling. Surface potential mapping was carried out at the same position under both dark and illuminated conditions, with a white light from an LED calibrated to one-sun intensity. To minimize noise, electrical potential profiles were averaged across all slow-scan lines. The difference between the illuminated and dark potential maps was then calculated, and the first derivative of this difference was used to derive the internal electric field distribution across the device.

## Results and Discussion

### Ligand Migration to the Top Interface

**Fig. 1:**
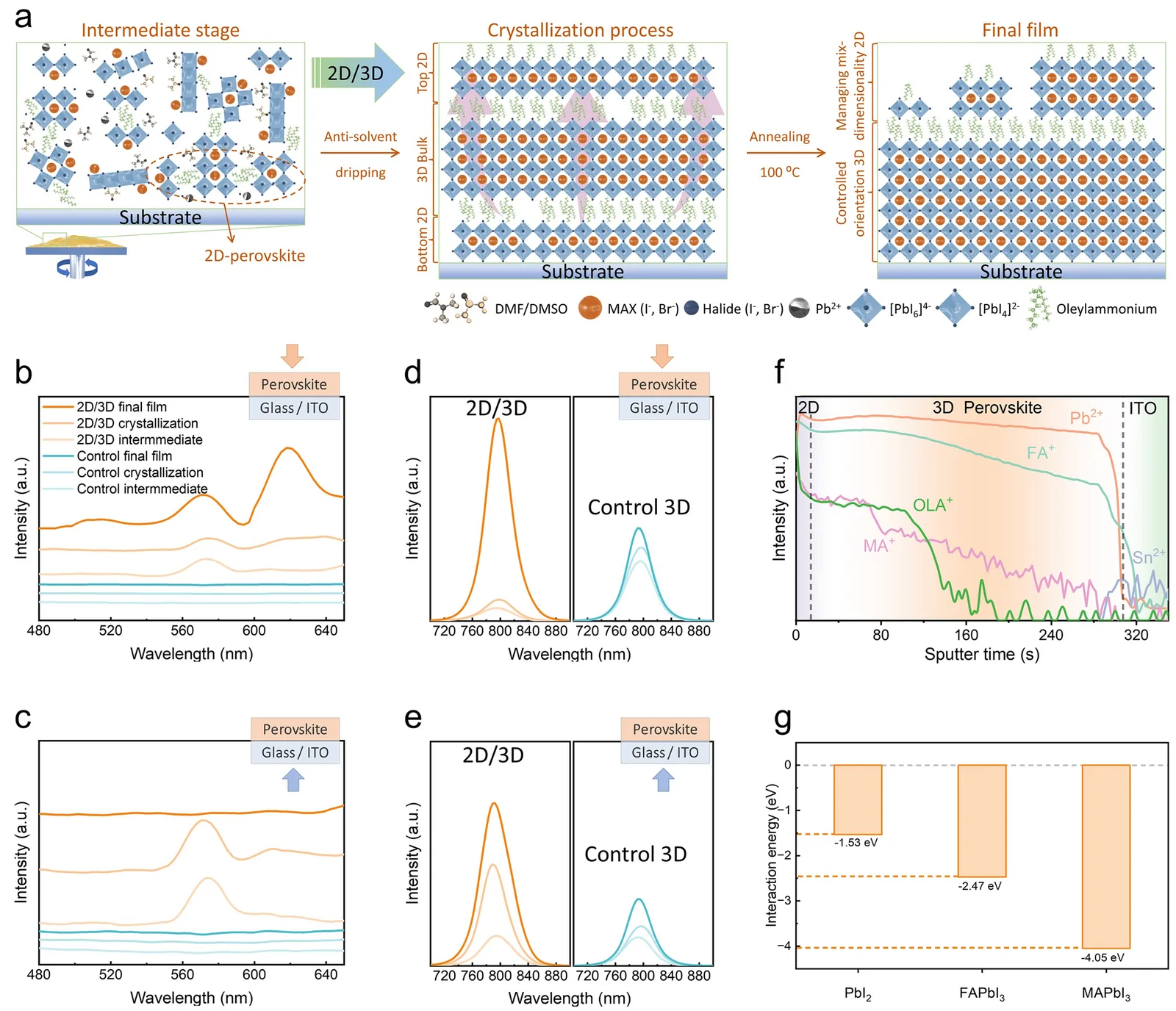
Migration of OlyAI ligand to the top surface. **a** Schematic representation of OlyA^+^ ligand migration to the top surface of 2D/3D perovskite film via ligand intercalation. PL spectra of control 3D perovskite and one-step 2D/3D perovskite at different fabrication stages measured from **b** top and **c** bottom surfaces, focusing on the region with 2D perovskite peaks. PL spectra of control 3D perovskite and one-step 2D/3D perovskite at different fabrication stages measured from **d** top and **e** bottom surfaces, focusing on the region with the main 3D perovskite peak. **f** TOF–SIMS analysis of the one-step 2D/3D perovskite film composition. **g** DFT binding energy of OlyAI on the (001) PbI_2_, (001) FAPbI_3_, and (001) MAPbI_3_ surfaces

We observed that the stoichiometric triple-cation lead iodide 3D perovskite (Cs_0.025_MA_0.075_FA_0.90_PbI_3_) suffers from uneven crystal orientation and high bulk defect density [28], which does not noticeably improve after 2D deposition via sequential post-treatment [32]. Such uneven crystal orientation and limited 2D penetration results in suboptimal 2D/3D bilayer formation, which fails to provide better passivation, and introduces charge transport limitations (Fig. S1). We incorporated OlyA^+^ ligand into the precursor solution in order to form a 2D/3D bilayer in situ, and to have better control over 3D perovskite crystallization. We expected the direct integration of OlyA^+^ ligand into 3D perovskite ink to create an effective growth template that regulates the crystal orientation of the underlying 3D perovskite at the intermediate stage. During subsequent annealing, 2D/3D bilayer formation is expected to occur at the top surface, as shown in Fig. 1a [5, 33].

We first conducted photoluminescence (PL) spectral analysis using a hyperspectral PL microscope (hyper-PL), measuring spectra from the top and bottom sides of 3D perovskite films formed with the addition of OlyA^+^ into the precursor ink in order to understand the ligand intercalation and spontaneous 2D/3D bilayer formation at the interfaces. Our investigation focused on three distinct conditions to probe the spatial distribution of 2D perovskite across 3D perovskite film (Figs. 1b–c and S2-S3): (i) intermediate phase, when the perovskite ink was freshly deposited onto the substrate, (ii) annealing for 1 min, and (iii) the final film. During the intermediate phase we observed a distinct peak at ~ 575 nm that corresponds to the 2D perovskite phase (*n* = 2) [5]. This signal was present at both the top and bottom of the film, where the 2D perovskite appeared to accumulate in an island-like structure across the surface (Figs. S2-S3). A weak signal for the 3D perovskite phase was detected at ~ 792 nm, indicating incomplete crystallization (Fig. 1d–e). Upon detecting the pronounced 2D signal even during intermediate phase, we conducted dynamic light scattering (DLS) analysis to reveal the mechanism of one-step 2D/3D formation in the precursor solution as shown in Fig. S4. DLS revealed that both control and OlyA^+^ precursor solutions are colloidal dispersions rather than true solutions. The control sample mainly consists of colloidal nanoparticles with sizes ranging from 1 to 8 nm that correspond to isolated (PbI_n_)^(n−2)−^ iodoplumbate complexes of various sizes, joined together by corner sharing Pb–I–Pb bonds into polyiodide frameworks [34, 35]. A small amount of colloidal particles with larger size ranging from 500 to 1000 nm also exists in the control solution with a total DLS intensity of 20% that corresponds to larger perovskite crystals. After the introduction of OlyAI we observed that the majority of colloidal particles have sizes ranging between 2000 and 6000 nm, which we attribute to the strong coordination between OlyA^+^ ligands and the perovskite framework.

During the crystallization stage, we found that the 2D signal from the top of the film becomes more uniform, with the *n* = 2 phase still dominant. However, the signal at the bottom of the film begins to transform, as depicted in Figs. 1c and S2-S3. In the final stage, the 2D *n* = 2 phase at the surface of the film evolved into a more prominent 2D *n* = 3 phase. Additionally, the enhanced 3D PL peak intensity suggests that incorporating OlyAI into the perovskite precursor ink slows down the crystallization process, a key factor in promoting high crystal quality [36]. We performed similar measurements on control 3D samples with no OlyAI addition and observed no sign of 2D perovskite formation below 700 nm, which corroborates our claim that OlyAI is directly responsible for in situ 2D/3D bilayer formation (Figs. 1d–e and S5). Additionally, the control sample exhibited greater spatial PL variation than the OlyA⁺ based film, indicating less uniform crystallization behavior.

We then further extended the scope of 2D ligands to short-chain phenethylammonium (PEA⁺) and iso-butylammonium (iBA⁺) iodides and conducted the same set of experiments, which have been extensively explored in the literature and consistently shown to form higher dimensionality of 2D phases [37, 38]. We observed that the PEA^+^ and iBA^+^ additives exhibit similar behavior at the intermediate phase of the fabrication process, which predominantly shows 2D perovskite formation with *n* = 2. However, the 2D signal diminishes during the crystallization process and is absent in the final film, as shown in Figs. S6–S7. This is because PEA^+^ and iBA^+^ are small alkyl ammonium ligands, which can easily penetrate into the crystal lattice of perovskite film or react with the A-site cation, thus breaking the 2D perovskite structures, as reported earlier [8, 10, 39].

We conducted time-of-flight secondary ion mass spectrometry (TOF–SIMS) measurements on complete perovskite films with half-device stack in order to obtain a depth profile and gain a deeper understanding of the migration and distribution of OlyA^+^ ligands during perovskite film formation, as shown in Fig. 1f. Our analysis revealed a significant change in the OlyA⁺ ion signal at the surface within 15 s of sputtering, indicating that most OlyA⁺ was concentrated at the top of the perovskite film. Additionally, a similar behavior was observed for MA⁺ ions, which were distributed throughout the film with higher concentrations near the top. Although OlyA⁺ was predominantly localized on the surface, trace amounts were detected throughout the bulk of the perovskite layer.

We also investigated 2D layer formation, and by extension OlyA^+^ distribution, using transmission electron microscopy (TEM), and mapped the spatial distribution of 2D perovskite layers in the 3D perovskite film (Figs. 1a and S8). The TEM images confirmed that the 2D perovskite formed by OlyA^+^ was primarily concentrated at the top of the perovskite layer. Interestingly, we also observed vertically aligned 2D layers along the grain boundaries, extending from the top toward the interior of the film. This finding correlated well with the TOF–SIMS results, where trace levels of OlyA^+^ cation were detected across the perovskite layer. These findings suggest that the 2D perovskite formed by OlyA^+^ acts as a surface passivation layer and penetrates into the grain boundaries, providing additional protection against ion migration. However, no 2D perovskite layer was found at the buried interface of the 3D perovskite film (Fig. S8). This one-step passivation mechanism at the surface of the perovskite and along the top side of grain boundaries is advantageous over the sequential post-treatment due to improved 3D perovskite quality.

We then performed a theoretical study using density functional theory (DFT) to investigate the surface-OlyAI interaction. The interaction energy with the (001) PbI_2_ surface (–1.53 eV) turns out to be lower than that with the (001) FAPbI_3_ (−2.47 eV) and (001) MAPbI_3_ (−4.05 eV) surfaces, as illustrated in Figs. 1g and S9a-c. The fact that OlyA^+^ binds much weaker to the (001) PbI_2_ surface than to the (001) MAPbI_3_ and (001) FAPbI_3_ surfaces reduces its intercalation and thus the peeling of layers from (MA,FA)PbI_3_ [6] in agreement with the experimental result that less n ≤ 2 2D perovskites than n > 2 2D perovskites are formed (Fig. 1b). The latter restricts the diffusion of MA^+^, which explains the observation that OlyA^+^ improves the thermal stability of (MA, FA)PbI_3_ [5]. During the intercalation, OlyA^+^ encounters more steric obstruction due to its larger size and larger region of negative electrostatic potential, see Fig. S9d-h, than 2-aminoindanium, a cation that induces 2D perovskite formation at the bottom of the sample [40]. As a result, the 2D perovskite formation is slower in the case of OlyA^+^ and therefore shifted to the top of the sample.

Furthermore, owing to its preferential interaction with the (001) MAPbI₃ surface, OlyA⁺ was found to accumulate predominantly at the top surface of the perovskite film. This observation is consistent with our TOF–SIMS measurements, which reveal that the majority of MA⁺ ions are also concentrated at the top and near-top surfaces (Fig. 1f) [33]. As we mentioned, the large size of the OlyA^+^ ligand could limit its penetration between [PbI_6_]^4−^ octahedral slabs, excluding it from the bulk during crystal growth. Therefore, we conclude that both the ligand size and perovskite composition are key factors governing the migration of OlyA^+^ to the top surface.

### 2D/3D Bilayer and Facet Engineering

**Fig. 2:**
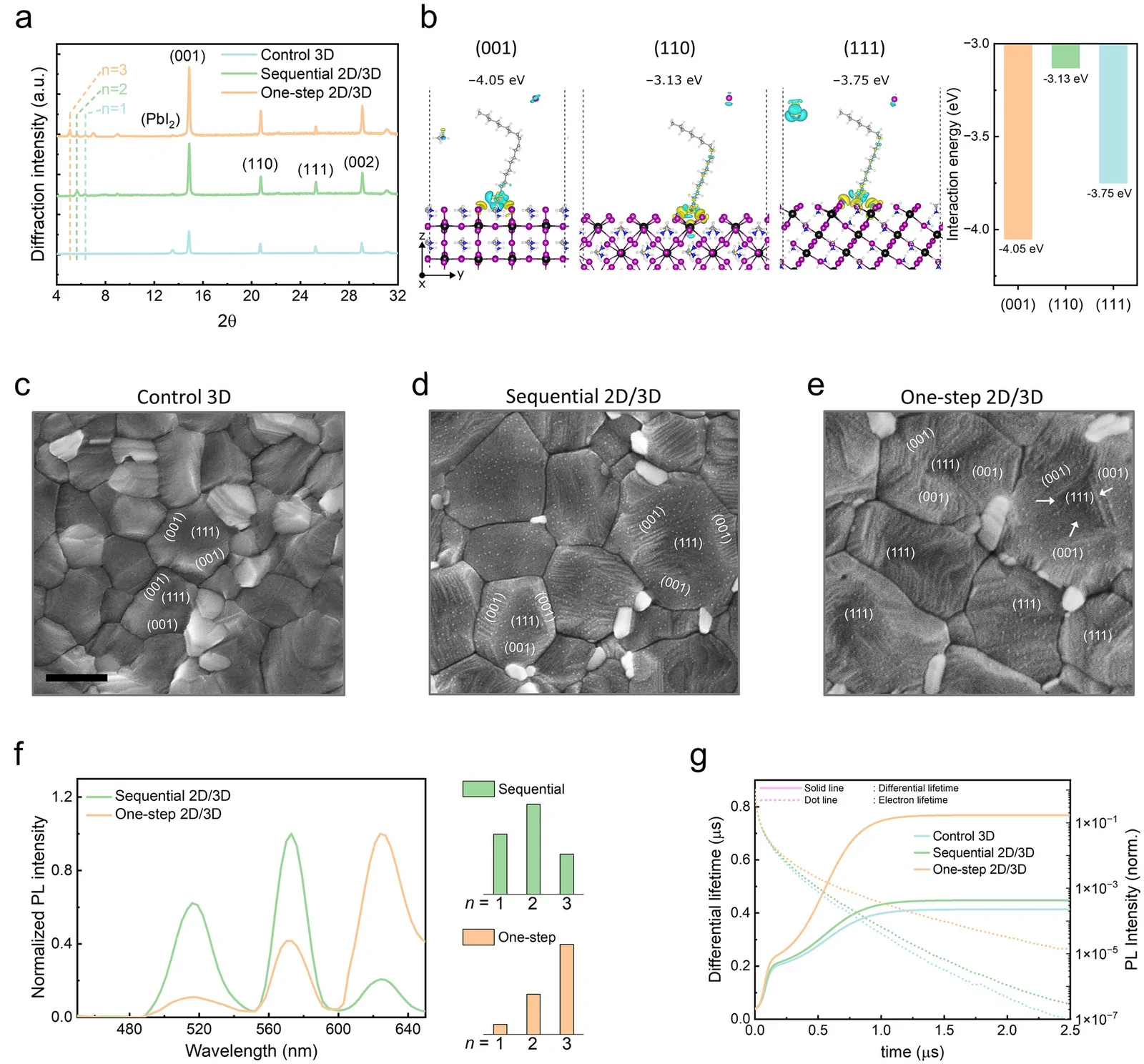
Crystallization of 2D at top interface (sequential 2D/3D vs one-step 2D/3D techniques). **a** XRD spectra of control 3D, sequential 2D/3D, and one-step 2D/3D perovskite films. **b** Structures and interaction energies of OlyAI on the I-terminated (001), (110) and (111) MAPbI₃ surfaces. Yellow and cyan isosurfaces represent charge accumulation and depletion, respectively. H = white, C = gray, N = blue, I = pink, and Pb = black. Top surface SEM images, defining the (001) and (111) facets for **c** control 3D, **d** sequential 2D, and **e** one-step 2D/3D perovskite films. The scale bar is 500 nm. **f** PL spectra of 2D perovskite films comparing sequential 2D/3D and one-step 2D/3D films. **g** Differential lifetime (solid lines, left axis) and photoluminescence intensity (dashed lines, right axis) of control 3D, sequential 2D/3D, and one-step 2D/3D perovskite films

Given the impact of the OlyA^+^ ligand on the perovskite film during the crystallization process, it is also important to investigate further how different processing methods influence the perovskite morphology. Here, as we previously reported, we will compare our one-step 2D/3D technique with the traditional post-treatment method to form a 2D/3D perovskite bilayer at the top-contact interface [5]. Throughout this study, the conventional post-treatment route for fabricating 2D/3D perovskite heterojunctions is denoted as “sequential 2D/3D”. We then studied the effect of the OlyA^+^ ligand on the crystallization growth of 3D perovskite using X-ray diffraction (XRD) and grazing-incidence wide-angle X-ray scattering (GIWAXS) spectroscopies following the intermediate phase, crystallization stage, and the final film formation. Figure 2a shows the different crystallinity between control 3D, sequential 2D/3D and one-step 2D/3D, indicating that the OlyA^+^ promotes facet-dependent crystallization growth along the (001) orientation while the (111) facet remains relatively unaffected compared to the control sample (Figs. 2a and S10a-c). During the annealing process in the one-step 2D/3D fabrication, we observed that the ratio of the (001) to (111) crystal planes increased by up to 1.8-fold, with only a slight increase thereafter (Fig. S11). This finding suggests that OlyA⁺ promotes the preferential formation of the (001) crystal orientation not only in the final film but already at the early stages of crystallization. The selective enhancement of (001) growth in the presence of OlyAI was then studied using DFT for I-terminated (001), (110), and (111) MAPbI_3_ surfaces. According to Fig. 2b, the interaction energy is − 4.05, − 3.13, and − 3.75 eV with the (001), (110), and (111) MAPbI_3_ surfaces, respectively, and the amount of redistributed charge agrees with this trend, suggesting that OlyA^+^ preferentially binds to and may promote the growth of (001) MAPbI_3_ facets, consistent with the experimental result (Fig. 2a). The selective enhancement of (001) growth in the presence of OlyA^+^ can be explained by the differences in surface polarity and electronic properties between (001) and (111) facets. The (001) facet is known to have a higher polarity and a more electron-rich environment, making it more favorable for interaction with ammonium groups [41]. This strong interaction can act as a templating effect, directing crystal growth along the (001) plane. In contrast, the (111) facet is less reactive toward ammonium-based additives and instead tends to interact with electron-rich groups or organic anions [41]. Additionally, the long alkyl chain of OlyA^+^ introduces steric hindrance, limiting its interaction with the (111) facet and reinforcing the preference for (001) orientation (Fig. S12). These findings highlight the critical role of facet-selective interactions in perovskite crystallization and provide valuable insights for controlling film morphology to enhance stability and performance.

Figure 2c–e shows scanning electron microscopy (SEM) images from the top view. The top view SEM images reveal well-defined polyhedral grain shapes for the control 3D, sequential 2D/3D, and one-step 2D/3D treatment perovskite films. Notably, the one-step 2D/3D treated sample exhibits larger and more uniform crystal morphology with suppressed triangular central facets, which is commonly attributed to the (111) orientation and more pronounced lateral facets corresponding to the (001) planes Figs. S12-S13 [25]. The preferential formation of the (001) orientation is particularly advantageous for optoelectronic applications [31, 41]. Indeed, the (001) direction is preferable for charge transport due to their fewer defect sites, and reduced nonradiative recombination compared to a film with randomly oriented crystals, which aligns well with our PL analysis above [41]. Thus, the improved crystal quality and preferred (001) orientation observed in the one-step 2D/3D film formation are expected to enhance the photovoltaic performance and stability of fabricated PSC devices.

In cross-sectional view, the control 3D shows a smaller and randomly oriented 3D perovskite grains with visible pinholes (Fig. S14). Conversely, perovskite films treated with the 2D/3D heterojunction technique exhibited significant improvements in crystallization. However, due to the steric hindrance of OlyA^+^ ligand, the penetration depth to regulate 3D perovskite is limited to the surface and near surface, leaving the lateral grain boundaries across the 3D perovskite, which is visible in the cross-sectional SEM image (Fig. S14). On the other hand, the one-step 2D/3D perovskite film exhibited a distinct vertical crystal orientation in the cross-sectional SEM image and visibly large grain size in the top SEM view, indicating more controlled growth of 3D perovskite film. We attribute this improvement in the crystallization growth to the interaction of long-chain ammonium ligand with the perovskite ink by anchoring to the A-site of the perovskite. The resulting vertical grain structure of 3D perovskite film confirms the successful role of the OlyA^+^ ligand as a template-guided agent in the crystallization process, which is advantageous for perovskite film quality [33]. We found that the incorporation of OlyAI provides additional passivation at the top surface of the perovskite film, and the long chain of the OlyA^+^ acts as a protective barrier against moisture ingress into the bulk of the 3D perovskite. We conducted water contact angle measurements and a water droplet test to evaluate the hydrophobicity of the films and their resistance against moisture ingress (Figs. S15-S16). The sequential 2D/3D and one-step 2D/3D heterojunction samples exhibited significantly higher moisture resistance, effectively blocking the infiltration of water molecules into the perovskite film. This confirms that OlyA^+^ ligands not only play a role in the crystallization dynamics and the 2D perovskite formation, but also serve as an efficient moisture barrier, enhancing the long-term stability of the perovskite films.

We further evaluated the impact of the deposition method on the structural and optical properties of 2D/3D perovskite by comparing the one-step technique with the conventional sequential technique. It is worth noting that there is a strong correlation between the quantum well effects and bandgap in 2D perovskites, which is directly related to the dimensionality of the 2D perovskite layers [42]. We then conducted PL spectroscopic measurements to map and explore the dependence of the dimensionality of 2D perovskite on the deposition method, as shown in Figs. 2f and S17. In the sequential 2D/3D films, we observed the presence of discrete dimensional 2D perovskite peaks with *n* = 1 (~ 520 nm), *n* = 2 (~ 575 nm), and *n* = 3 (~ 620 nm). PL analysis confirmed that most of the emission originated from the *n* = 2 and *n* = 1 phases, indicating that 2D perovskites with lower dimensionality are dominant in the sequential process. In contrast, the one-step 2D/3D perovskite films exhibit a predominance of the *n* = 3 phase, with smaller contributions from *n* < 3. This suggests that the one-step method favors the formation of 2D perovskites with higher dimensionality, which results in an improved energy alignment with the 3D perovskite/ETL interface.

We then illustrate the energy-level diagram for the 3D perovskite and 2D perovskites with different dimensionality (n = 1, 2, 3) prepared with OlyA^+^ ligands and compare them with charge transport layers. Valence band maximum (VBM) of these layers were obtained from photoelectron spectroscopy in air (PESA) and ultraviolet photoelectron spectroscopy (UPS) measurements, and CBM levels were calculated from the optical bandgap using PL (Figs. S18-S21). We observed a type I band alignment between the 3D and 2D perovskites, where the CBM of the 2D layer is elevated and the VBM is lowered relative to the 3D perovskite (Fig. S18). The insulating nature and weak electronic coupling of the OlyA^+^ ligand induce a strong quantum confinement effect on the 2D perovskite layer, resulting in a widened bandgap and poor energy level matching with the ETL layer for efficient electron extraction. As the dimensionality of 2D perovskite layer increases, the quantum confinement effect progressively weakens due to the increase in inorganic perovskite thickness. Further, a higher dimensionality of the 2D layer allows for a more delocalized distribution of the charge carrier wavefunction, resulting in upward shift of the VBM and a deeper CBM [43, 44]. This shift brings the CBM of the 2D perovskite layer closer to the CBM of the C_60_, thus the one-step 2D/3D could improve energetic alignment between the perovskite and the ETL layer compared to 2D/3D which was deposited sequentially, needed for optimal electron extraction (Fig. S18). Additionally, variations in work function (WF) were observed among the samples. The control 3D exhibited a less uniform surface and a higher WF, while sequential 2D/3D and one-step 2D/3D demonstrated lower WF values, closer to the CB. Notably, one-step 2D/3D displayed a more uniform WF distribution compared to the other samples, suggesting improved electronic properties for potential optoelectronic applications (Fig. S22). This behavior can also be attributed, in part, to the large dipole moment of the OlyA^+^ ligand, which induces vacuum level shifts at the interface [45, 46]. Such dipolar effects become more pronounced in lower-dimensional phases, where electronic coupling is limited, contributing to the mismatch with ETL. As the dimensionality increases, the influence of the dipole is moderated by enhanced electronic delocalization, leading to more favorable energy alignment for electron extraction.

The presence of OlyAI was further confirmed by X-ray photoelectron spectroscopy (XPS) (Fig. S23), which verified the absence of additional impurities in the system. Our analysis revealed that the concentration of OlyAI at the perovskite surface is higher when using the sequential deposition technique compared to the one-step method. This suggests that an excess of OlyAI accumulates in the sequential process, aligning with our hypothesis (Figs. S24-S25). Furthermore, the Pb 4*f* core-level spectrum exhibits peak broadening, possibly indicative of 2D phase formation in both the sequential and one-step methods (Fig. S26).

We further conducted time-resolved photoluminescence (TRPL) measurements and differential lifetime analysis of the perovskite films, which provides key insights into charge carrier dynamics and interfacial recombination (Fig. 2g). By examining TRPL decays (dashed lines, right y-axis), we observed that one-step 2D/3D had the longest decay time, followed by sequential 2D/3D and control 3D samples. The control sample exhibited the shorted decay time, suggesting additional nonradiative recombination pathways. To understand these differences in more detail, we calculated differential lifetimes from the TRPL decays [47] (solid lines, left y-axis). The one-step 2D/3D sample exhibited the highest plateau differential lifetime (i.e., saturation lifetime), suggesting it had the lowest interfacial and Shockley–Read–Hall recombination rates. The sequential 2D/3D had the next highest saturation lifetime, followed by the control 3D sample. Additionally, the one-step 2D/3D sample had the steepest slopes in the ~ 100 to ~ 250 ns and 250 ns to 1 µs regions, suggesting that it had the fastest charge transfer (CT) rates to the SAM/ITO electrode. Thus, the one-step 2D/3D sample had the lowest interfacial recombination and the fastest CT rates, followed by the sequential 2D/3D, and lastly the bulk 3D samples.

### Device Performance and Electrical Properties

**Fig. 3:**
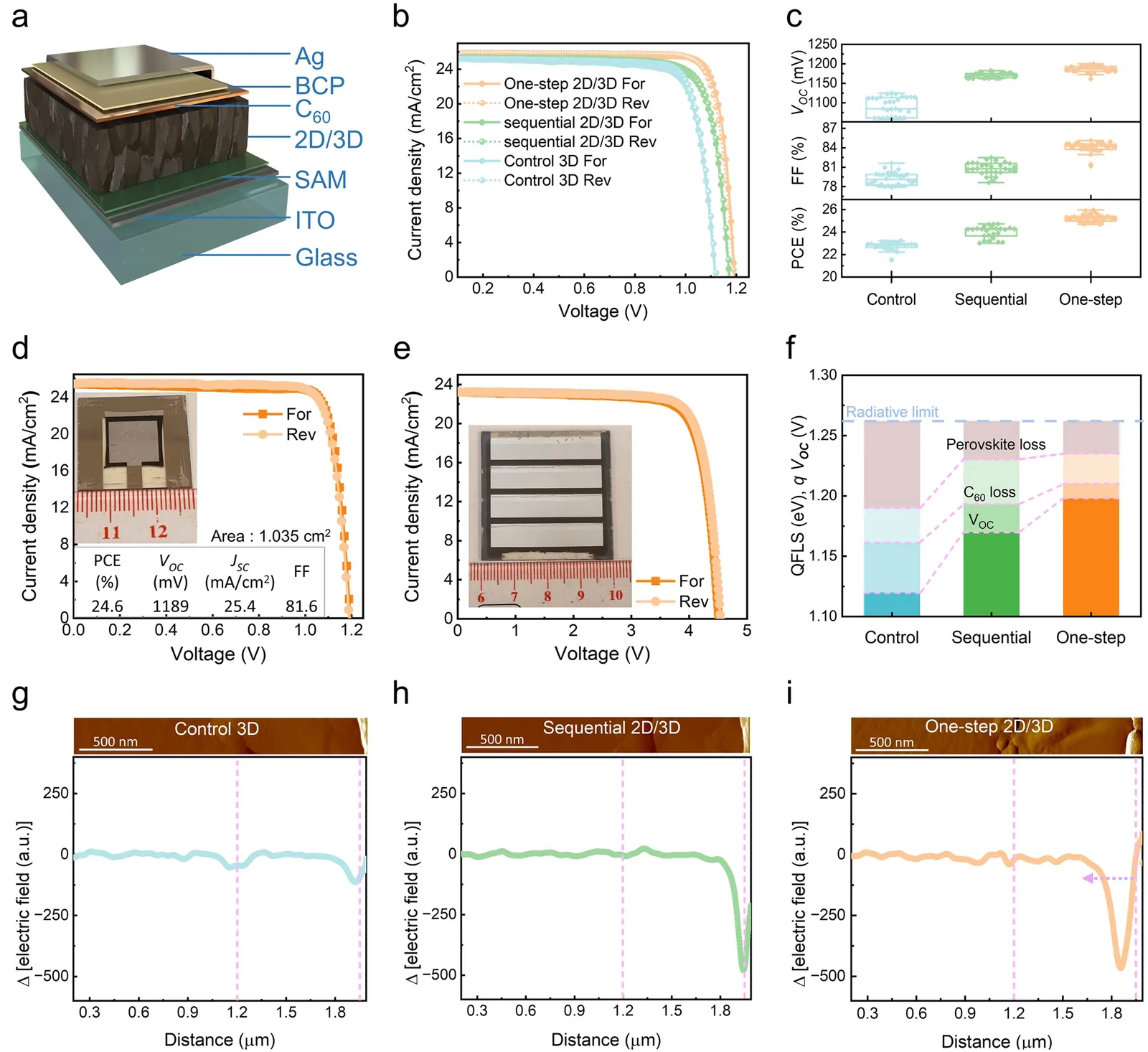
Device performance. **a** Device architecture of inverted perovskite single-junction solar cells. The device performance of control 3D, sequential 2D/3D, and one-step 2D/3D perovskite solar cells with **b** *J-V* characteristics of champion cells, and **c** device statistics. *J-V* characteristics of the one-step 2D/3D perovskite champion device with an increased area of **d** 1.035 cm^2^ and **e** 6.8 cm^2^. **f** *V*_OC_ loss analysis for control 3D, sequential 2D, and one-step 2D/3D perovskite solar cells. Electric field distribution through both interfaces from the C_60_ electron transport layer to the glass/ITO using cross-sectional KPFM measurements for **g** control 3D, **h** sequential 2D, and **i** one-step 2D/3D perovskites

We then transferred the one-step 2D/3D strategy using OlyA^+^ to our single-junction inverted PSCs following the structure shown in Fig. 3a. As a result, the devices fabricated with 1.55 eV band gap perovskite (Cs_0.025_MA_0.075_FA_0.90_PbI_3_) by one-step 2D/3D method achieved a champion PCE of 26.22% (open-circuit voltage (*V*_OC_) = 1.198 V, short-circuit current density (*J*_SC_) = 25.89 mA cm^−2^ and fill-factor (FF) = 85.12) with the integrated J_SC_ from EQE was 25.61 mA cm^−2^ (Fig. S27). This performance was significantly higher compared to control device (PCE = 23.2%, *V*_OC_ = 1.12 V, *J*_SC_ = 25.3 mA cm^−2^, FF = 81.1) and represented a ~ 1.5% improvement over the devices with sequential 2D/3D perovskite (PCE = 24.3%, *V*_OC_ = 1.17 V, *J*_SC_ = 25.4 mA cm^−2^, FF = 81.1) (Fig. 3b and Table S1). As shown in Fig. 3c, the main improvements in the device performance were attributed to V_OC_ and FF, while the *J*_SC_ showed only minor differences. This indicates that the one-step 2D/3D perovskite fabrication method effectively regulates the perovskite/ETL interface. Additionally, we also applied the one-step 2D/3D fabrication method to wider bandgap perovskite, observing a similar trend in device performance improvements over control 3D devices (Fig. S28). Besides modifying the device configuration, we also applied this one-step technique to various ligands. Specifically, we used PEAI as commonly used as Ruddlesden–Popper (RP) perovskites and propylenediammonium dihydroiodide (PDAI) for Dion–Jacobson (DJ) perovskites (Fig. S29) [48, 49]. Both systems showed improvements across all device parameters. However, it is worth noting that the formation of 2D/3D structures with different ligands should be investigated on a ligand-to-ligand basis (Fig. S6). This demonstrates that the one-step 2D/3D technique has a wider applicability, suggesting its potential for development in tandem devices.

We then measured stabilized power output (SPO) at a fixed maximum power point (MPP) voltage for devices prepared by one-step 2D/3D method and observed a stabilized SPO within 10 s. This result highlights a minimal hysteresis effect, consistent with the J-V characteristics. The device maintained SPO under continuous illumination for approximately 60 min, demonstrating that the one-step 2D/3D method significantly suppresses defect formation caused by prolonged illumination (Fig. S30). We also transferred the one-step 2D/3D method to larger substrates with active area of 1.0 and 6.8 cm^2^ using spin-coating technique to assess the quality of in situ grown 2D layers over the large area. The resulting devices showed PCE of ~ 24.6% and 21.1% for 1.0 and 6.8 cm^2^ devices, respectively, highlighting the scalability of the one-step 2D/3D fabrication method (Fig. 3d–e).

We then quantified the quasi-Fermi level splitting (QFLS) of control 3D, sequential 2D/3D, and one-step 2D/3D perovskite films from absolute PL measurements to investigate the quality of 2D/3D heterojunction and highlight their radiative limit throughout the perovskite film (Fig. S31). The result demonstrates that both sequential 2D/3D and one-step 2D/3D films exhibit a maximum QFLS value of approximately 1.24 eV, which is ~ 0.05 eV higher than that of the control 3D film (~ 1.19 eV). However, the sequential 2D/3D technique possesses uniformity issues which typically occur in the spin coating process, especially for ultra-thin film deposition, resulting in a broad statistical distribution on QFLS histogram analysis (Fig. S32a). This spatial variation in the QFLS values was also observed after depositing C_60_ on top of the perovskite (Fig. S32b). Contrastingly, the one-step 2D/3D method can suppress nonradiative recombination after the deposition of C_60_ much better compared to sequential 2D/3D method, indicating the importance of the former as a passivation for defects in PSCs. Developed from the QFLS, we then conducted a V_OC_ loss analysis, combining with the theoretical radiative limit, and device V_OC_ (Fig. 3f). Based on the perovskite band gap and the theoretical radiative limit, all three devices can attain a maximum theoretical V_OC_ of ~ 1.26 eV. However, in practice perovskites deposited with either sequential 2D/3D or one-step 2D/3D methods have much higher defect tolerance resulting in relatively high bulk QFLS (1.230 and 1.235 eV, respectively) compared to control 3D perovskite (1.190 eV). Moreover, perovskite prepared by one-step 2D/3D method can better suppress losses associated with the deposition of C_60_, which can also be observed from practical V_OC_ of devices where one-step 2D/3D showed 1.19 V, while sequential 2D/3D and control 3D samples stayed at 1.17 and 1.12 V, respectively.

We also identified the electronic properties of the perovskites prepared by control 3D, sequential 2D/3D, and one-step 2D/3D methods at the device level using space-charge-limited current (SCLC) measurements. For the electron-only devices in SCLC measurements we employed the device structure of ITO/SnO_2_/Perovskite/C_60_/Ag, while for hole-only devices we used ITO/PTAA/Perovskite/Spiro-OMeTAD/Ag. The dark current–voltage sweep of electron-only devices unveiled that the one-step 2D/3D perovskite exhibited a smaller trap-filled limited voltage (V_TEL_ = 0.12 V) compared to sequential 2D/3D (V_TFL_ = 0.15 V) and control 3D (V_TFL_ = 0.25 V) samples (Fig. S33a). This indicates that defects in the one-step 2D/3D film are filled more rapidly by the injected charge. A similar trend can be observed with hole-only devices (Fig. S33b). This underscores the ability of the one-step 2D/3D method to efficiently suppress bulk defect density by regulating the 3D perovskite crystallization and serving as an effective 2D passivation layer. However, it is worth noting that SCLC limits on extracting the absolute value of trap density state due to the presence of mobile ions [50, 51]. To further confirm the charge recombination dynamics, we then performed an analysis using transient photovoltage (TPV) and transient photocurrent (TPC). As shown in Fig. S34a-b, the control 3D device exhibits the shortest decay lifetime ($$\tau _{{ave}}$$ = 24.319 µs), reflecting accelerated carrier recombination kinetics. This rapid decay can be ascribed to a higher density of trap-assisted recombination centers and bulk defect states. In contrast, the OlyA⁺ 2D/3D significantly prolonged the photovoltage decay, with $$\tau _{{ave}}$$$$\tau _{{ave}}$$ increasing to 37.505 and 52.412 µs for the sequential 2D/3D and one-step 2D/3D, respectively. Furthermore, in the TPC measurements, the one-step 2D/3D device exhibits a faster photocurrent decay, indicating more efficient charge extraction and transport. Combined with the slower photovoltage decay, these results suggest that the one-step 2D/3D device experiences reduced carrier recombination and improved charge carrier transport dynamics.

We further conducted cross-sectional KPFM measurements to investigate the built-in electric field within the device under both positive and negative voltage bias (Figs. 3g–i and S35). As shown in Fig. 3g, the control 3D device exhibits only a minor change in the surface potential near the ETL, indicating low charge extraction quality. In contrast, devices incorporating OlyA⁺ display a pronounced enhancement in the electric field, with comparable behavior observed in both the sequential and one-step 2D/3D configurations (Fig. 3h–i). This suggests that the presence of OlyA⁺ effectively strengthens the built-in potential and facilitates more efficient charge extraction. Notably, the one-step 2D/3D device shows a slightly extended potential tail toward the perovskite bulk region, which can be attributed to the accumulation of OlyA⁺ near the upper surface. This extended potential distribution for one-step device at the electron-contact side helps form an interfacial energy barrier that effectively suppresses hole backflow while promoting electron extraction, consistent with the observed improvement in overall device performance and the electron charge extraction or transfer.

### Stability Analysis

**Fig. 4:**
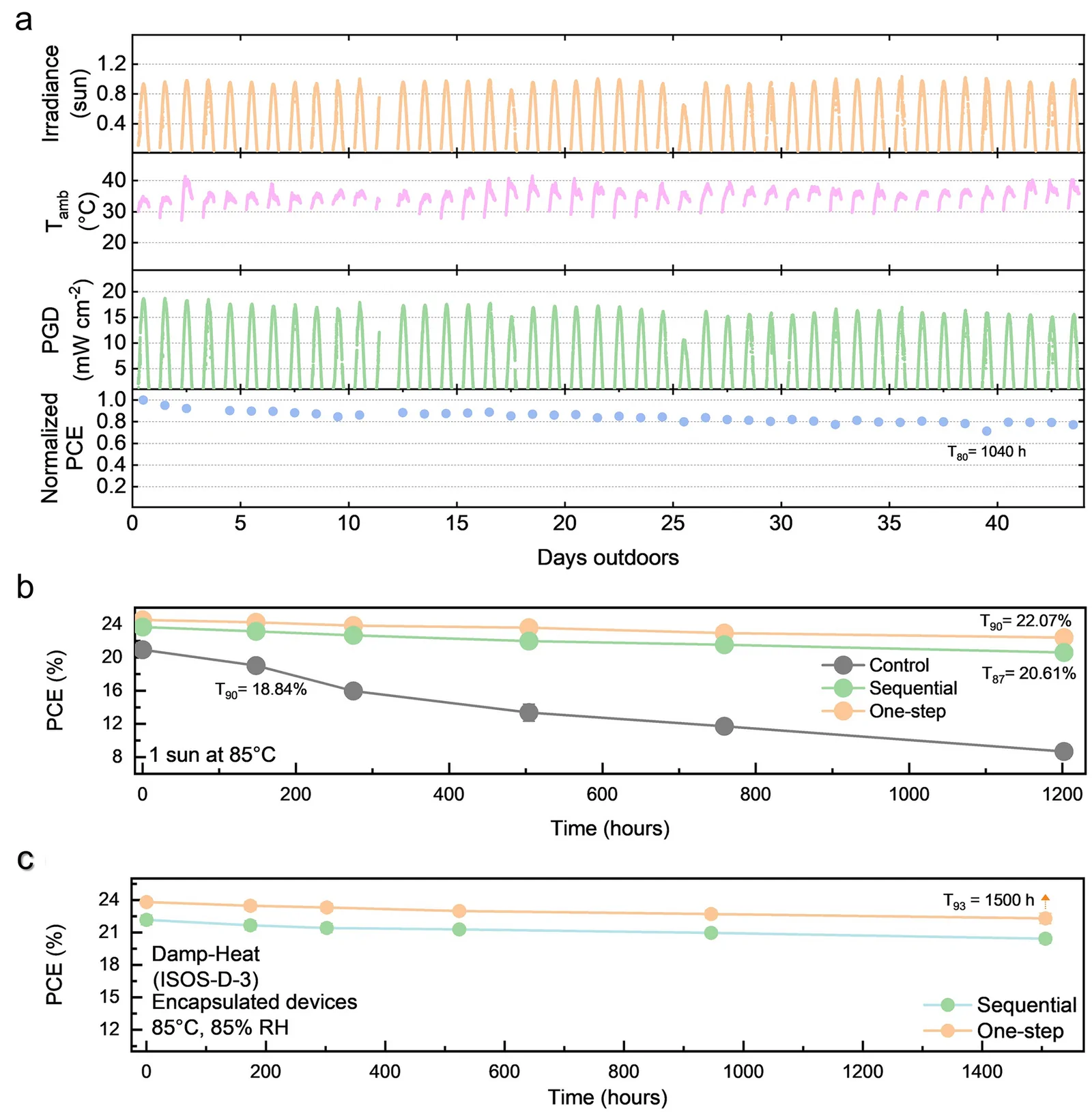
Device stability. **a** Outdoor test under ISOS-O-2 protocol of an encapsulated single-junction device prepared by one-step 2D/3D method under real-world conditions in Thuwal, Saudi Arabia. PCE evolution for sequential 2D/3D and one-step 2D/3D PSCs under **b** photothermal, and **c** damp-heat testing conditions

We then evaluated the long-term stability of PSCs following the International Summit on Organic Photovoltaic Stability (ISOS-O-2) protocols, as shown in Fig. 4a. The device was encapsulated and tested under natural sunlight in Thuwal, Saudi Arabia (22°18′17.0"N, 39°06′30.1"E) on a fixed south-facing rack with a tilt angle of 25°. To mitigate Ag penetration and moisture diffusion, bathocuproine (BCP) was replaced with an atomic layer-deposited SnO_2_ as a buffer layer. The resulting one-step 2D/3D device demonstrates a PCE retention of approximately 80% from their initial value (T_80_) after 1040 h of continuous operation. To assess possible structural evolution upon environmental exposure, we then compared the as-fabricated and post-outdoor-tested samples. The PL analysis reveals a slight decrease in the lower-dimensional (n = 1) 2D phases, accompanied by an increase in the n = 2 and n = 3 phases. These results suggest that OlyA^+^ preferentially stabilizes higher-dimensional 2D configurations while exerting minimal influence on the 3D perovskite lattice, indicating that OlyA^+^ does not perturb the bulk 3D framework (Fig. S36a-b). Additionally, thermogravimetric analysis (TGA) was performed to confirm that the OlyA^+^ ligand retains 99% of its mass up to 200 °C (Fig. S37). We then evaluated the photothermal stability of 2D OlyAI, we conducted accelerated aging tests under continuous 1-sun illumination at 85 °C (Fig. 4b). The initial PCEs of the devices were observed to be 20.94% for the control device, 23.67% for the sequential 2D/3D, and 24.53% for the one-step 2D/3D configuration. The results reveal that the control 3D device experienced significant degradation, losing up to 50% of its PCE after 700 h. In contrast, the device sequentially treated with the 2D layer exhibited improved operational stability, showing only a 10% loss of its initial PCE after ~ 800 h. Interestingly, the one-step 2D/3D device demonstrated further enhancement, retaining 90% of its initial efficiency for up to 1200 h. We then conducted damp-heat testing (85 °C and 85% relative humidity under open-circuit conditions) on encapsulated devices, revealing that those fabricated using the sequential technique retained 92% of their initial PCE after 1500 h, while the one-step 2D/3D devices retained 93% with a higher overall PCE (Fig. 4c). Notably, the one-step approach not only demonstrated comparable stability but also delivered a higher initial PCE compared to the sequential method. Additionally, we performed the reverse-bias induced device degradation, showing that the improved stability with one-step 2D/3D by increasing the breakdown current from − 4 V for control, − 6.2 V for the sequential 2D/3D, and − 6.9 V for the one-step 2D/3D device (Fig. S38).

## Conclusion

We demonstrate a spontaneous in situ strategy for fabricating 2D/3D bilayer perovskite heterojunction by incorporating long-chain OlyAI directly into the perovskite precursor ink. This one-step approach enables simultaneous crystallographic orientation control, favoring the (001) facet of 3D perovskite film and serving as a template for 2D perovskite growth, resulting in more efficient interfacial passivation, while also overcoming the limitations of conventional sequential 2D/3D deposition methods. The resulting inverted PSCs exhibit high power conversion efficiencies (up to 26.22% for 0.1 cm^2^ and 24.6% for 1 cm^2^) and outstanding long-term stability under damp heat and outdoor testing conditions, with 90% and 80% retention of their initial PCE. Our findings highlight the crucial role of ligand-guided crystallization and interface engineering in advancing scalable, stable, and efficient perovskite solar cell technologies.

## Supplementary Information

Below is the link to the electronic supplementary material.

Supplementary file1 (DOCX 9799 KB)
