# Supplementary material for: One-Step Formation of 2D/3D Perovskite Heterojunction via Ligand Intercalation and Facet Engineering for Efficient Perovskite Solar Cells
Source: Nanomicro Lett. 2026 Feb 9;18:240. doi: 10.1007/s40820-025-02058-8 (PMC12886586; doi:10.1007/s40820-025-02058-8)
Supplement: Supplementary file 1 — Supplementary file1 (DOCX 9799 KB) [file 40820_2025_2058_MOESM1_ESM.docx]

Supporting Information for

**One-Step Formation of 2D/3D Perovskite Heterojunction via Ligand Intercalation and Facet Engineering for Efficient Perovskite Solar Cells**

Drajad Satrio Utomo^1,2^, Yanping Liu^3^, Andi Muhammad Risqi^4^, Mohammed Ghadiyali^1^, Imil Fadli Imran^1,2^, Rakesh Rosan Pradhan^1^, Shynggys Zhumagali^1,2^_,_ Sofiia Kosar^1,2^, Vladyslav Hnapovskyi^1,2^, Christopher E. Petoukhoff^2^, Hao Tian^5^, Xiaoming Chang^1,2^, Badri Vishal^2^, Adi Prasetio^1,2^, Anil Reddy Pininti^1,2^_,_ Marco Marengo^1,2^, Ahmed Ali Said^1,2^, Aleksandra Oranskaia^1^_,_ Jongbeom Kim^4^, Chuanxiao Xiao^5,6^, Frédéric Laquai^1,7^, Thomas D Anthopoulos^1,8^, Udo Schwingenschlögl^1^, Sang Il Seok^4,^*, Randi Azmi^1,2,3^*, Stefaan De Wolf^1,2,^*

^1^Physical Science and Engineering (PSE), King Abdullah University of Science and Technology (KAUST), Thuwal 23955-6900, Kingdom of Saudi Arabia

^2^Center for Renewable Energy and Storage Technologies (CREST), King Abdullah University of Science and Technology (KAUST), Thuwal 23955-6900, Saudi Arabia

^3^School of Science and Engineering, The Chinese University of Hong Kong (Shenzhen), Shenzhen, Guangdong 518172, P. R. China

^4^School of Energy and Chemical Engineering, Ulsan National Institute of Science and Technology (UNIST), 50 UNIST-gil, Eonyang-eup, Ulju-gun, Ulsan, 44919 Republic of Korea

^5^Ningbo Institute of Materials Technology and Engineering, Chinese Academy of Sciences, Ningbo City, P. R. China

^6^Ningbo New Materials Testing and Evaluation Center Co. Ltd, Ningbo City, P. R. China

^7^Physical Chemistry and Spectroscopy of Energy Materials, Ludwig Maximilians University Munich, Butenandtstraße 11 (E), D-81377 Munich, Germany

^8^Henry Royce Institute, Photon Science Institute, Department of Electrical and Electronic Engineering, The University of Manchester, Manchester M13 9PL, UK

*Corresponding authors. E-mail: [seoksi@unist.ac.kr](mailto:seoksi@unist.ac.kr) (Sang Il Seok); [randiazmi@cuhk.edu.cn](mailto:randiazmi@cuhk.edu.cn) (Randi Azmi); [stefaan.dewolf@kaust.edu.sa](mailto:stefaan.dewolf@kaust.edu.sa) (Stefaan De Wolf)

**S1 Method**

***Device Stability Measurement***. All the stability measurements were done under an encapsulated sample. The device was firstly sandwiched between two solar glass sheets (3.2 mm thick) with thermoplastic polyurethane, which serves as the encapsulant, and butyl rubber (HelioSeal PVS101) as the edge sealant. An additional electrode using indium-tin alloy (GS120, MBR electronics GmbH) was soldered onto the sample electrode. Furthermore, the full stack undergoes lamination in a commercial laminator (Ecoprogetti Ecolam 5) at 120° C for 20 minutes. Following lamination, the metal ribbons are soldered to electric cables with MC4 connectors, which connect the cell to the measurement set-up outdoors. The devices are installed at the KAUST outdoor testing facility (22°18'17.0"N, 39°06'30.1"E) on a fixed south-facing rack with a tilt angle of 25°. The solar cells are kept under maximum power point tracking (MPPT) at 1-minute intervals, while IV scans are conducted every 10 minutes to monitor photovoltaic parameters degradation (Jsc, Voc, FF). In parallel with device performance, environmental conditions such as ambient temperature and irradiance are continuously monitored. The damp-heat test was performed in a dark environmental chamber set to constant temperature and humidity (85 °C/85% RH) for approximately 1,400 h. After the devices were allowed to cool for a few minutes in ambient air, they were periodically measured inside a glove box filled with N2. For the photothermal test, the encapsulated cells were exposed to full 1-sun illumination and maintained at a steady temperature of 85 °C on a hot plate in ambient air, where the relative humidity exceeded 50%. Measurements of the devices were then taken periodically until reaching 1,200 h.

***Sample Characterization***. The photoluminescence peak and mapping were performed using hyperspectral fluorescence microscopy (Hyper-PL, Photon, etc. IMA) equipped with a low-working distance 20x magnification microscope and 3nm spectral resolutions. The observation area was captured by using 405 nm lasers on the active area (440 µm $\times$ 320 µm) with wavelength range from 450 nm – 900 nm. Then the lasers switched to 532 nm to measure Quasi-fermi level splitting (QFLS). For the QFLS measurement, the laser excitation power was calibrated to the photon flux under standard solar spectrum conditions at AM1.5G 100 mW cm^-2^ [S1]. The data were obtained following the procedure used in the previous experiment and the reference literature [1]. Then the QFLS were further analyzed using home-build MATLAB code, extracting the 150 µm $\times$ 150 µm from the observation area following the equation:

$$J\sim a (E)\frac{E^{2}}{e\frac{E-\Delta\mu}{kT}-1}$$

Where $J,a,\Delta\mu,E, and kT$ are the emitted photon flux density, absorbance, Quasi-fermi level splitting, photon energy, and thermal energy. The time-of-flight secondary ion mass spectrometry (ToF-SIMS) measurements (Model TOF–SIMS 5, ION-TOF GmbH, Germany) were conducted using pulsed primary ions from a Cs^+^ (1 keV) liquid-metal ion gun sputtering and a Bi^3+^ pulsed primary ion beam for the analysis. Powder X-ray diffraction (XRD) was performed on a Bruker D2 Phaser diffractometer using Cu Kα radiation (λ = 1.54 Å), operating at 30 kV and 10 mA. Measurements were carried out at room temperature (25 °C) over a 2θ range of 3–35°, with a step size of 0.02° and a 2-second count time per step. A linear position-sensitive detector (PSD) with a 2θ opening angle of 3.78° was used to collect the diffraction data.

GIWAXS measurements were performed at the 6D UNIST-PAL beamline of the Pohang Accelerator Laboratory (PLS-II), Korea. The X-ray source, derived from a bending magnet, was monochromatized to an energy of 18.986 keV (corresponding to a wavelength of 0.6530 Å) using a double-crystal monochromator. A two-dimensional CCD detector (MX225-HS, Rayonix) was employed to capture the scattering patterns. Precise sample alignment was achieved using a five-axis motorized stage integrated within the GIWAXS measurement chamber. The sample-to-detector distance was maintained at 242.58 mm, and the setup was calibrated using a standard LaB₆ reference material (NIST SRM660b). An incident angle of approximately 0.2° was chosen to enhance surface sensitivity and to obtain detailed crystallographic information of the perovskite thin films.

The interaction of OlyAI (NH_3_^+^-group pointing towards the surface) with the I-terminated (001) PbI_2_, (001) FAPbI_3_, (001) MAPbI_3_, (110) MAPbI_3_, and (111) MAPbI_3_ surfaces was studied for stoichiometric slabs of 4 × 4 × 4 perovskite cells, facilitating negligible ion-ion interaction, with a vacuum layer of at least 25 Å thickness. These calculations were performed using the Vienna ab-initio simulation package [S2] with the generalized gradient approximation of Perdew, Burke, and Ernzerhof [S3], the Grimme van der Waals correction [S4], the Bengtsson dipole correction [S5], a plane-wave cutoff energy of 450 eV, and Γ-point integration. The stopping criterion of the structural relaxation was 0.01 eV/Å for the Hellmann-Feynman forces. The interaction energy was calculated as difference between the total energies without and with OlyAI on the surface. Charge accumulation and depletion isosurfaces were calculated at an electron density level of 0.001 e/Å^3^ by the Bader method. Electrostatic potential maps were calculated at an electron density level of 0.001 e/Å^3^ by relaxing OlyA^+^ and 2-aminoindanium in vacuum as well as OlyA^+^ in implicit solvents (dimethylsulfoxide, n,n-dimethylformamide, and anisole) through a polarizable continuum model [S6, S7] using Gaussian16 [S8], the B3LYP functional, and a 6-311++G**(d,p) basis set.

The dynamic light scattering (DLS) was conducted using a Zetasizer Nano ZS particle size analyser from Malvern Instruments Ltd. Scanning electron microscopy (Auriga, Zeiss) observed the top surface and cross-sectional morphology. The magnification was set around 10.000- 40.000 and 5.5 mm working distance. The 5 kV electron beam was used during the measurement. The water contact angle on the surface was measured using KRUSS- The drop shape analyzer DSA 100 system automatically. TRPL signals were acquired on perovskite/SAM/ITO samples using a gated intensified charge-coupled device (iCCD) camera system, triggered by a picosecond laser source (Innolas picolo AOT MOPA, 532 nm, 800 ps pulse duration, 3 kHz repetition rate). The excitation fluence was maintained at 17.94 µJ·cm⁻², with a beam diameter of 2.55 mm. The excitation was directed onto the perovskite film side. The sample’s emission was collected through a system of lenses and projected onto the input slit of a spectrograph (SpectraPro SP-2150i from Princeton Instruments). Detection was accomplished using a gated iCCD camera (Pi-MAX 4 from Princeton Instruments), synchronized with the laser pulses to capture emission dynamics. The gate width was fixed to 2.88 ns for all gate delays.

Topographic and surface potential measurements, were employed using a Digital Instruments Multimode AFM system (Veeco Metrology Group) with a Bruker scanning probe microscope head. A conductive SCM-PIT V2 AFM probe was used, fabricated from antimony-doped silicon (resistivity: 0.01–0.025 Ωcm) and coated with reflective Pt-Ir (platinum-iridium). The rectangular-shaped cantilever had a nominal resonant frequency of 75 kHz and a spring constant of 4 N/m. Prior to work function (WF) measurements, the probe tip was calibrated using a gold reference substrate to ensure accurate WF determination.

The photoelectron spectroscopy measurements were conducted using a multi-probe UHV system (ScientaOmicron) operating at a base pressure of 10^-9^ – 10^-10^ mbar, equipped with Argus CU electron energy analyzer. The UPS measurements were carried out using a vacuum ultraviolet (VUV) He (1) discharge lamp (21.2 eV) (HIS-13 FOCUS) equipped with a copper attenuation aperture to limit the VUV intensity and surface charging. The samples were biased with 10 V. The photoelectrons were collected at an angle of 80° between the sample and analyzer, with a normal electron take-off angle. The constant analyzer pass energy (CAE) was 2 eV for the valence band region and for the secondary electron cutoff. All measurements were carried out in electrical contact to the analyzer without charge neutralization. All external light was eliminated during measurements by covering port windows. The work function of the analyzer was calibrated to a clean metallic Au reference sample. Analysis of UPS data was conducted in Origin by linear extrapolation to identify the secondary electron cutoff and valence band onset. XPS was carried out in the same spectrometer, equipped with a monochromatic Al Ka X-ray Omicron XM1000 X-ray source (hv = 1486.6 eV) operating at a power of 390 W. The high-resolution (survey) spectra were collected at a constant pass energy of 15 eV (50 eV) at electron take off angle perpendicular to the surface. The spectra were analyzed with Casa XPS software. The individual peak envelopes were fitted with a GL(30) line-shape with a Tougaard-based background function.

Transient photovoltage (TPV) and transient photocurrent (TPC) measurements were conducted using a Fluxim PAIOS system. The devices were illuminated with a square-shaped light pulse of varying intensities (5–200 mW cm⁻²) for 200 μs.

**Supplementary Figures and Tables**


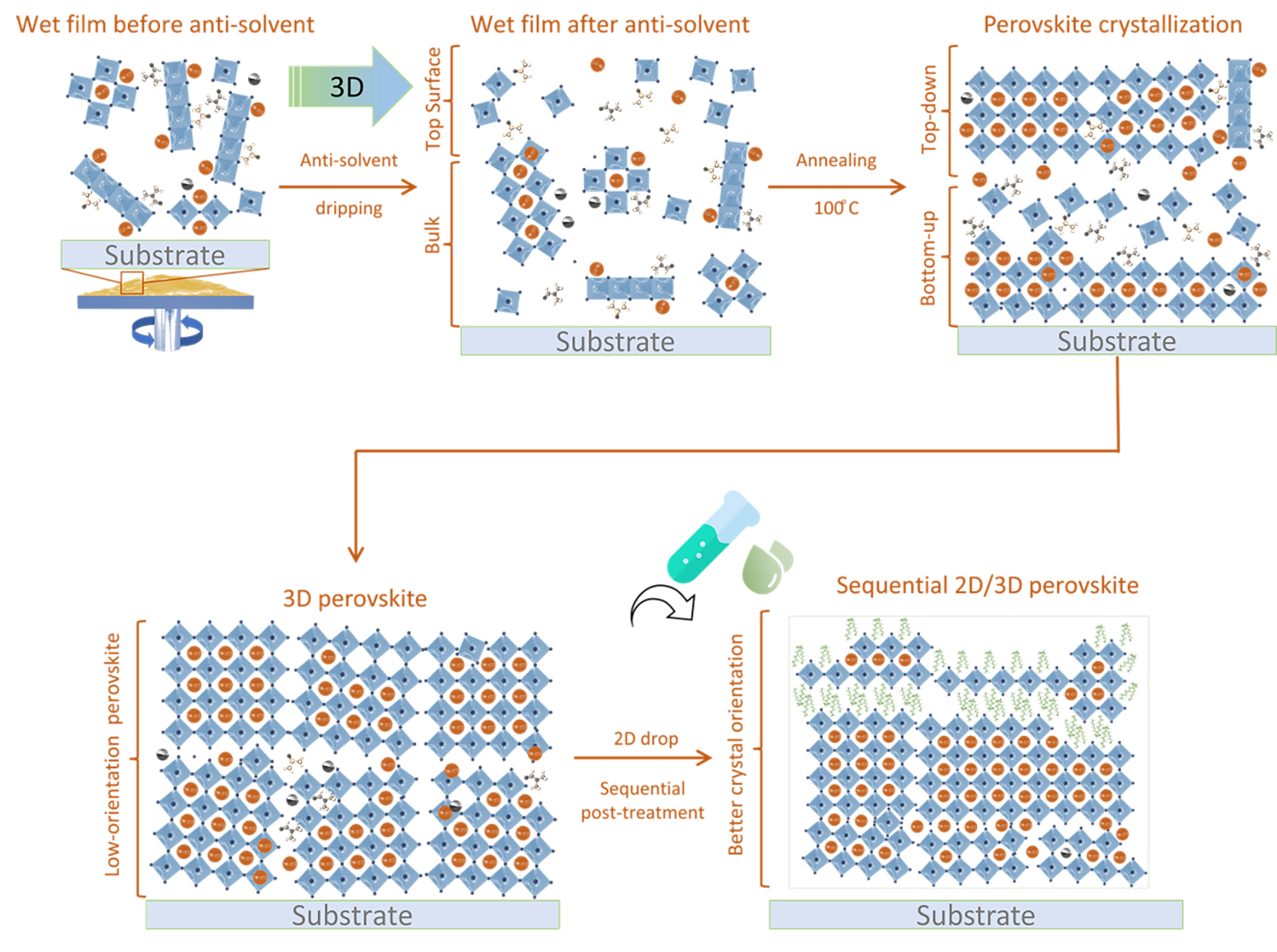


**Fig. S1** Schematic representation of ammonium ligand deposited via sequential technique on the 3D perovskite surface


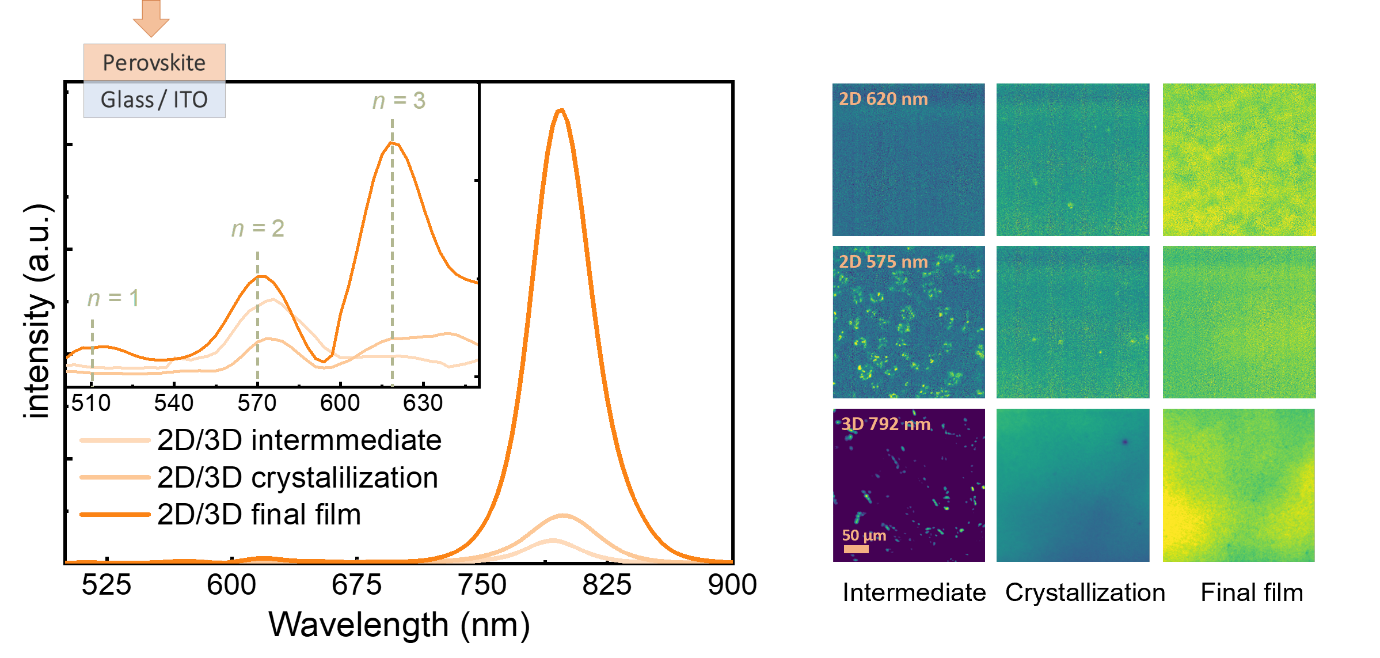


**Fig. S2** PL evolution of 2D/3D bilayer perovskite formed by oleylammonium iodide measured from the top side


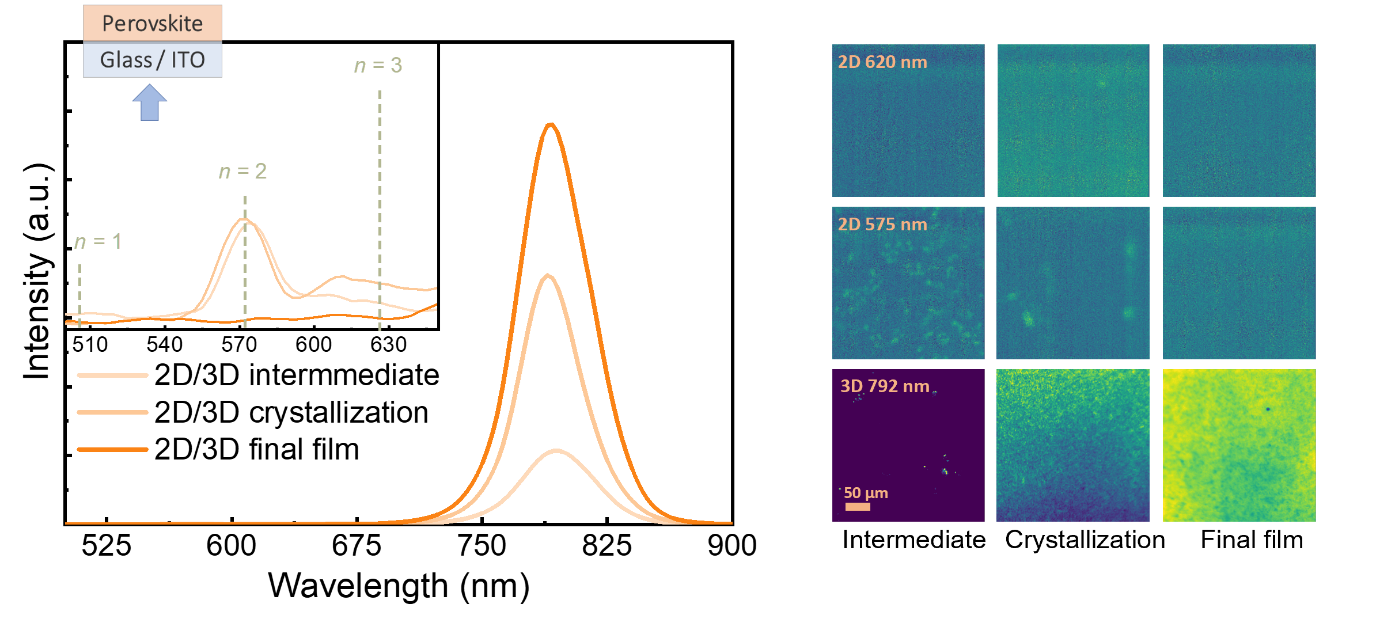


**Fig. S3** PL evolution of 2D/3D bilayer perovskite formed by oleylammonium iodide measured from the bottom side


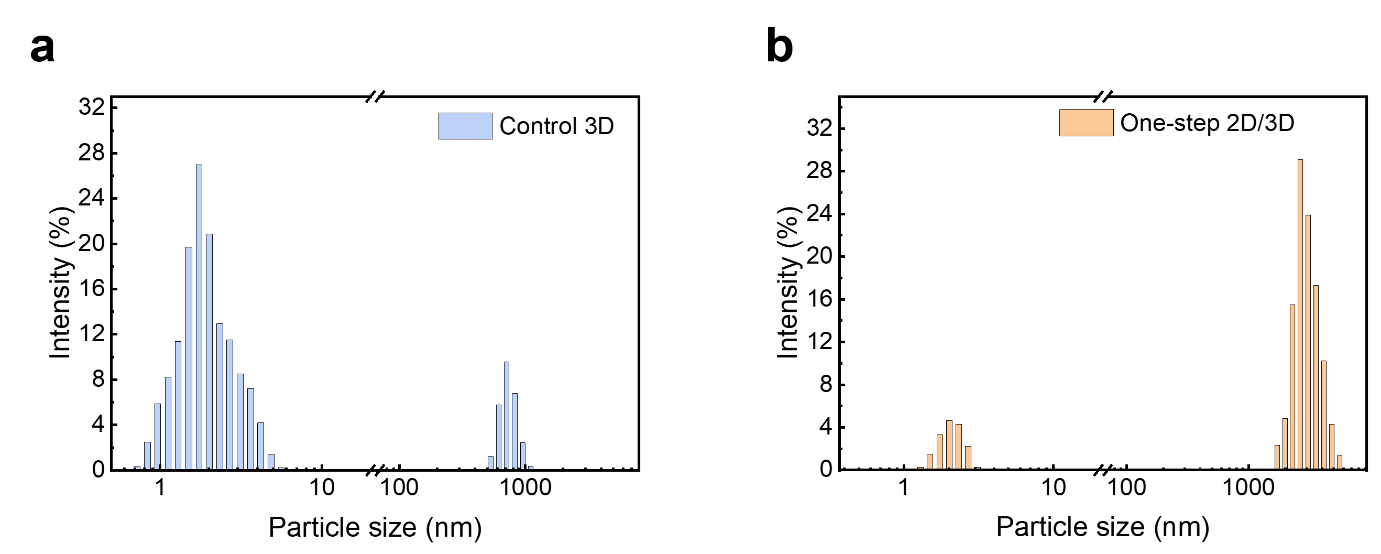
**Fig. S4** DLS result of the perovskite ink using (Cs_0.025_MA_0.075_FA_0.90_PbI_3_) mixture as (**a**) control and (**b**) OlyAI ammonium ligand

**
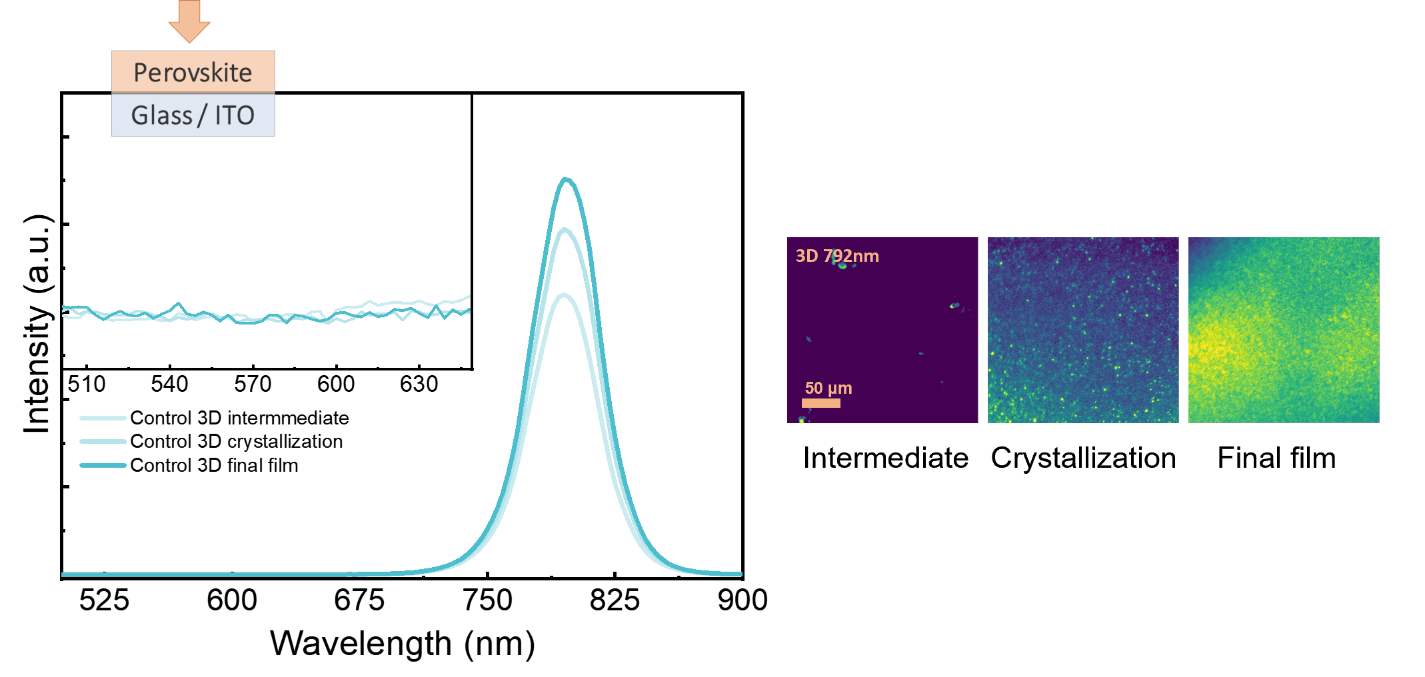
Fig. S5** Hyperspectral PL from the top side of control sample film


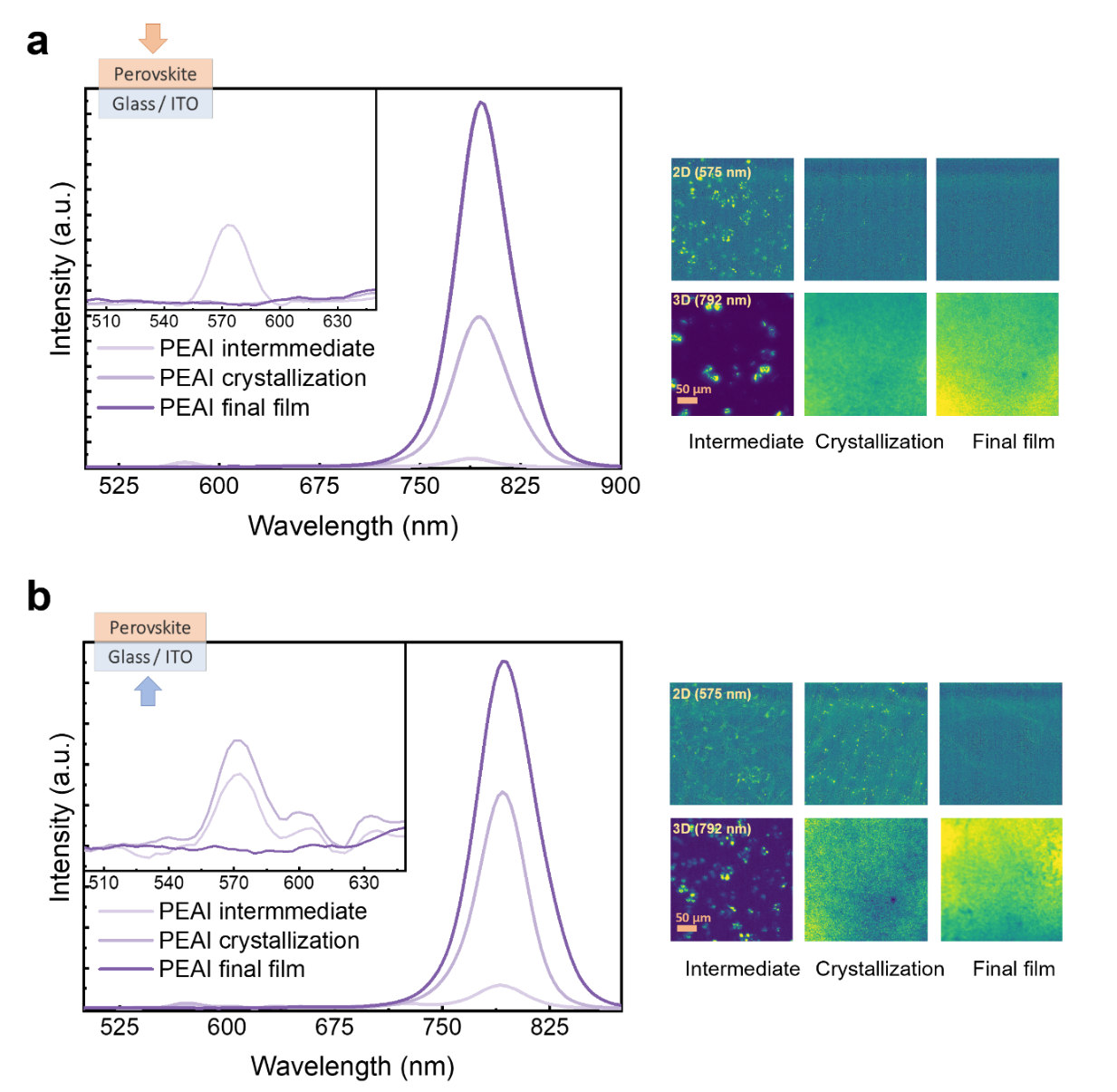


**Fig. S6** 2D/3D PL evolution of perovskite based on phenethylammonium iodide additive from (**a**) top side, and (**b**) bottom side

**
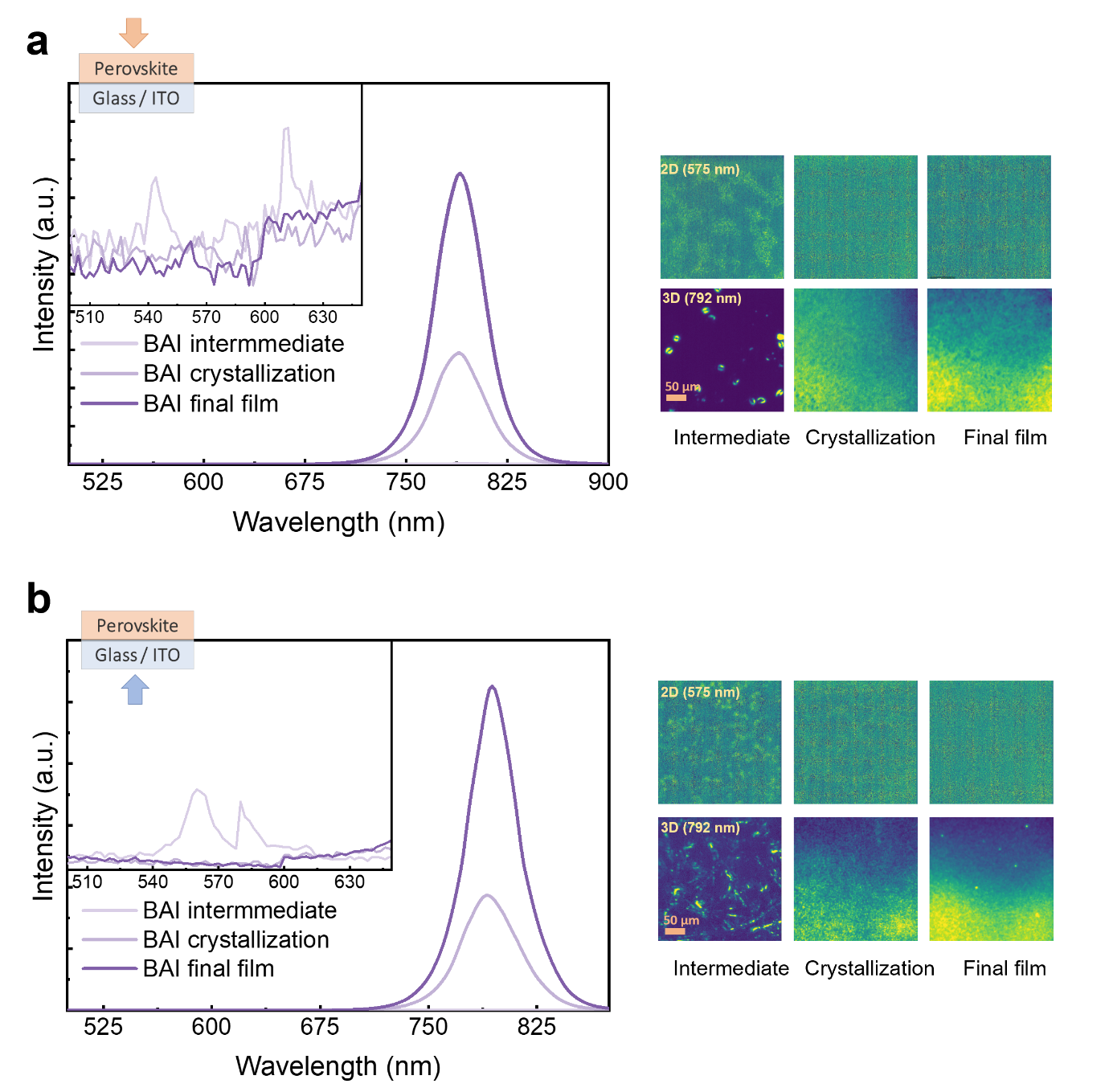
Fig. S7** 2D/3D PL evolution of perovskite based on iso-butylammonium iodide addtive from (**a**) top side, and (**b**) bottom side


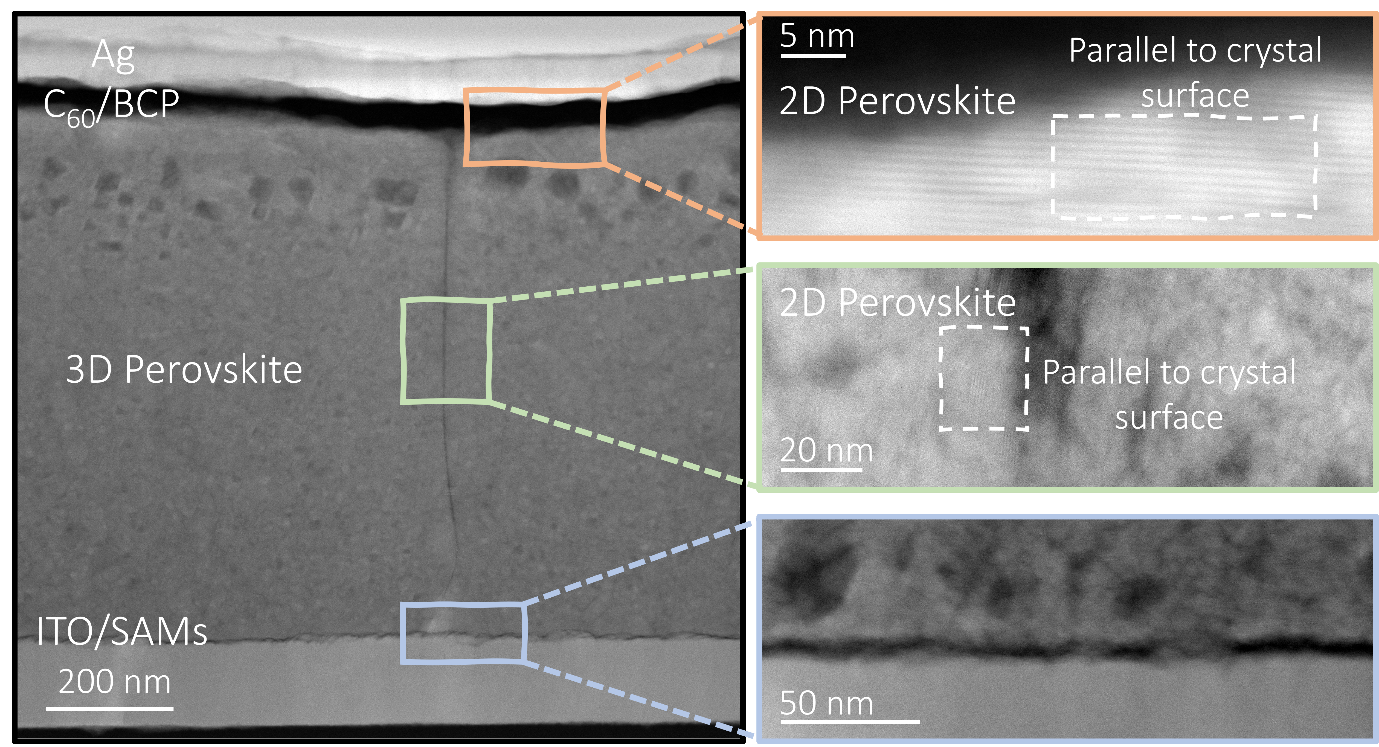


**Fig. S8** TEM images of one-step 2D/3D perovskite using oleylammonium iodide ligands

**
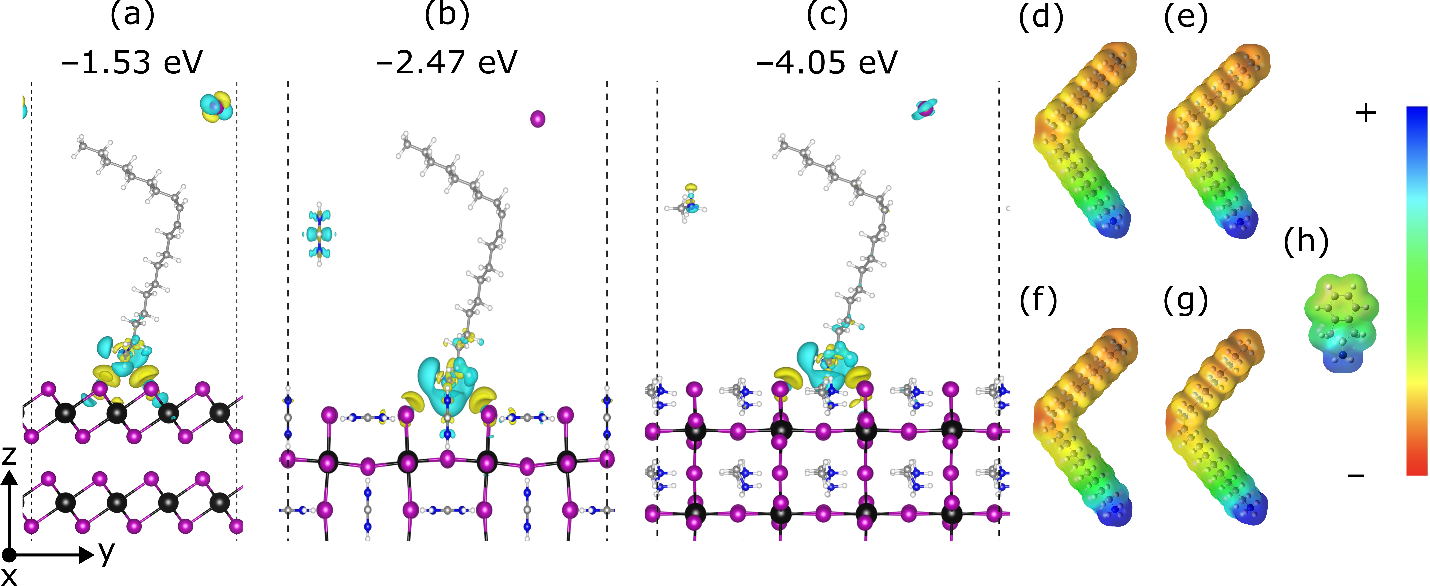
Fig. S9** Structures and interaction energies of OlyAI on the I-terminated (**a**) (001) PbI_2_, (**b**) (001) FAPbI_3_, and (**c**) (001) MAPbI_3_ surfaces. Yellow and cyan isosurfaces represent charge accumulation and depletion, respectively. Electrostatic potential maps of OlyA^+^ in (**d**) vacuum, (**e**) dimethylsulfoxide, (**f**) n,n-dimethylformamide, and (**g**) anisole and of (**h**) 2-aminoindanium in vacuum. H = white, C = grey, N = blue, I = pink, and Pb = black


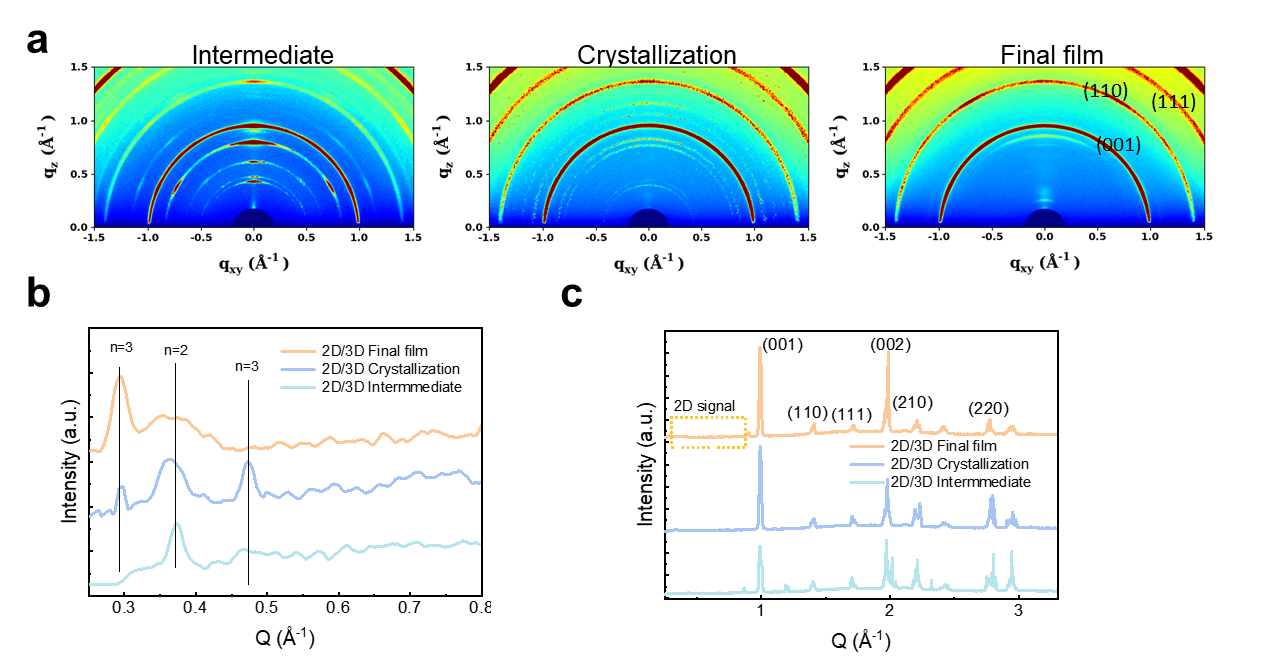


**Fig. S10** GIWAXS measurement of one-step 2D/3Dmigration from top surface (**a**) pattern, (**b-c**) line cut signal from 2D region and whole region, respectively


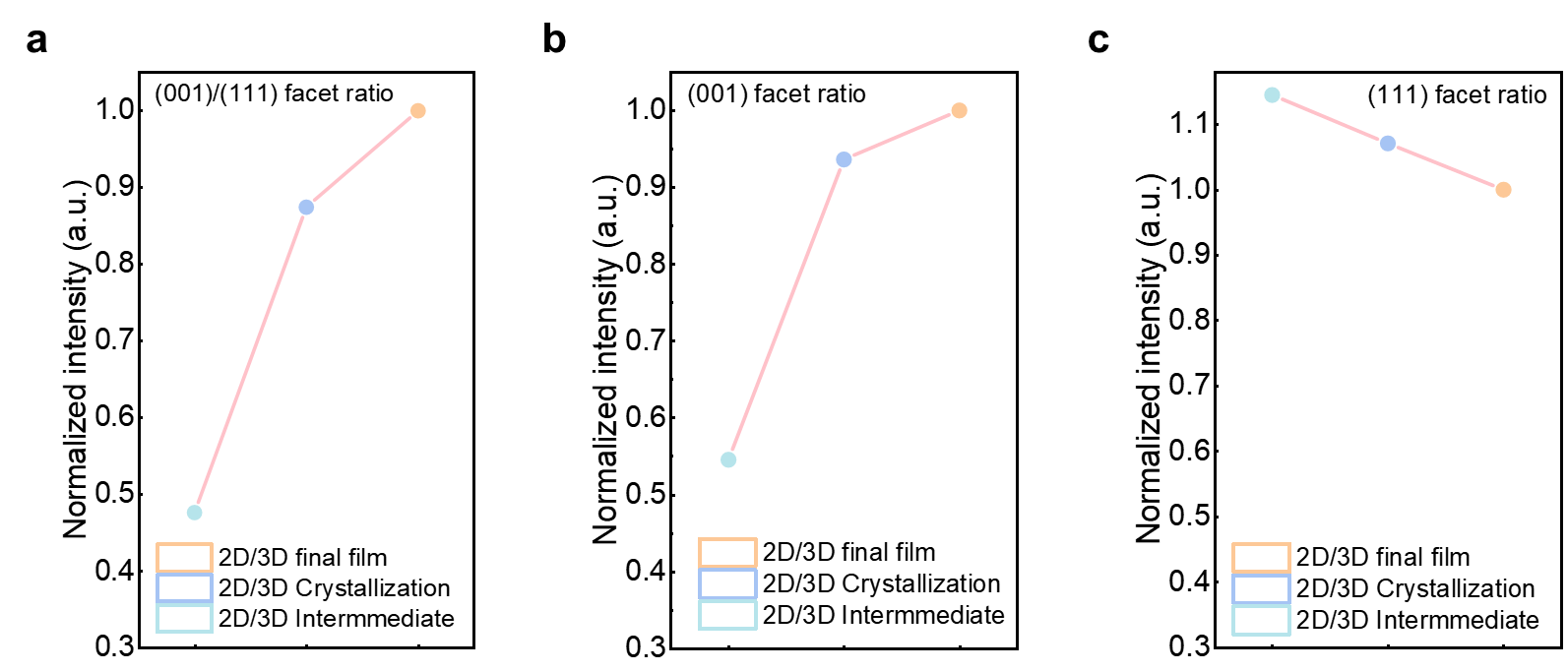


**Fig. S11** (**a**) (001)/(111), (**b**) (001), and (**c**) (111) GIWAXS facet ratio from azimuthal integration data as a function of annealing condition in one-step 2D/3D perovskite film


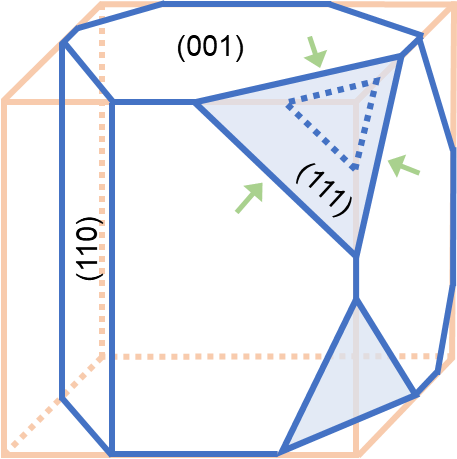


**Fig. S12** Illustration images of the perovskite surface for (001) and (111) facets, highlighting the growth of (001) facets as 2D/3D template


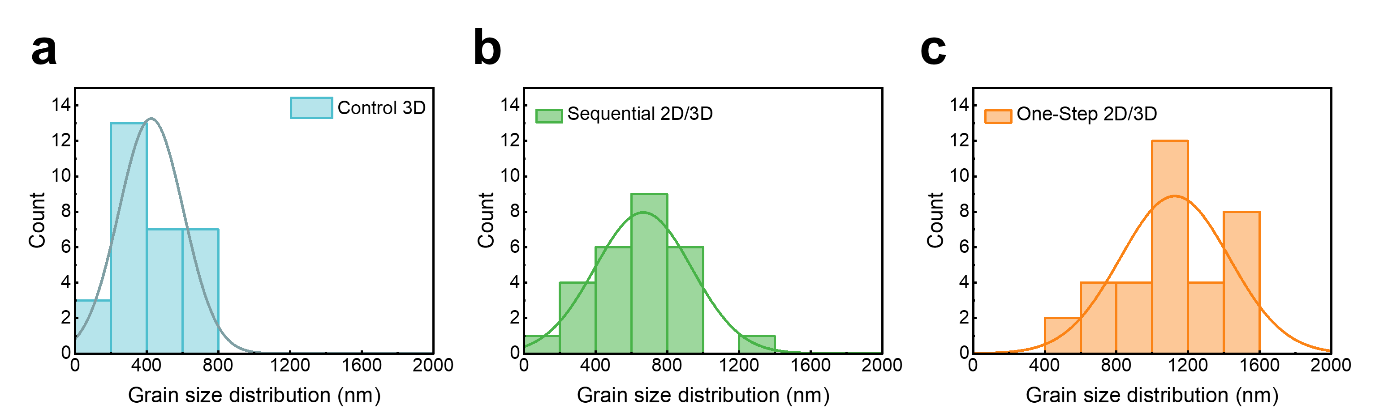


**Fig. S13** Grain size analysis from top view SEM for (**a**) control 3D, (**b**) sequential 2D/3D, and (c) one-step 2D/3D


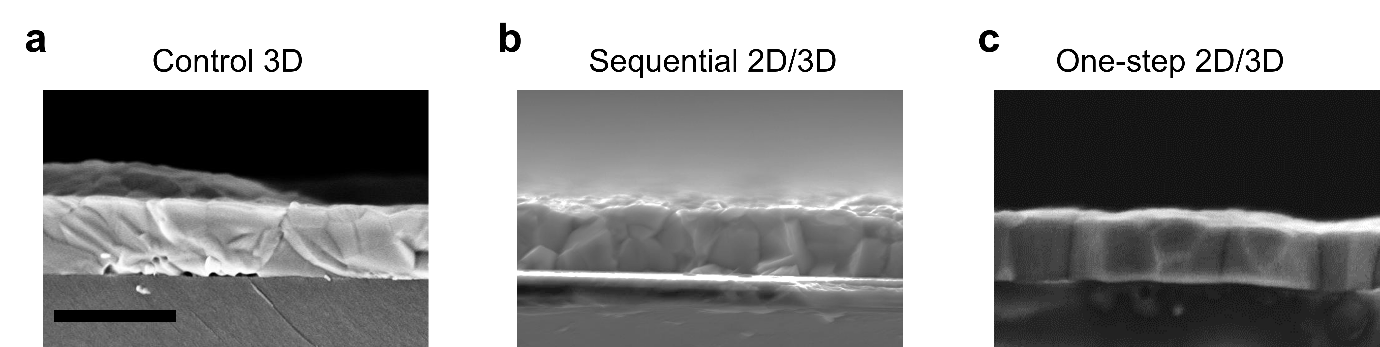


**Fig. S14** Cross-sectional SEM of the perovskite film for (**a**) control 3D, (**b**) sequential 2D/3D, and (**c**) one-step 2D/3D, scale bar 2 µm


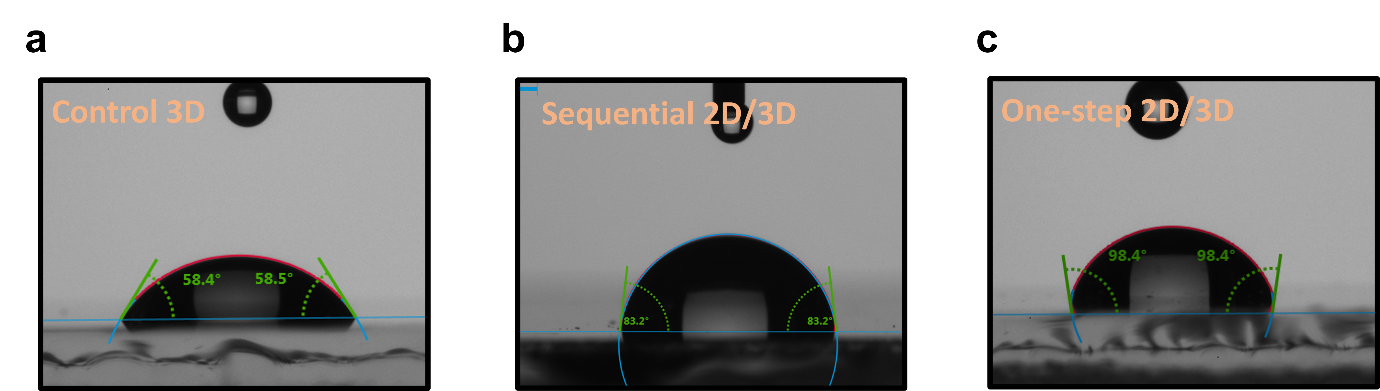


**Fig. S15** Water contact angle of (**a**) control 3D, (**b**) sequential 2D/3D, and (**c**) one-step 2D/3D perovskite


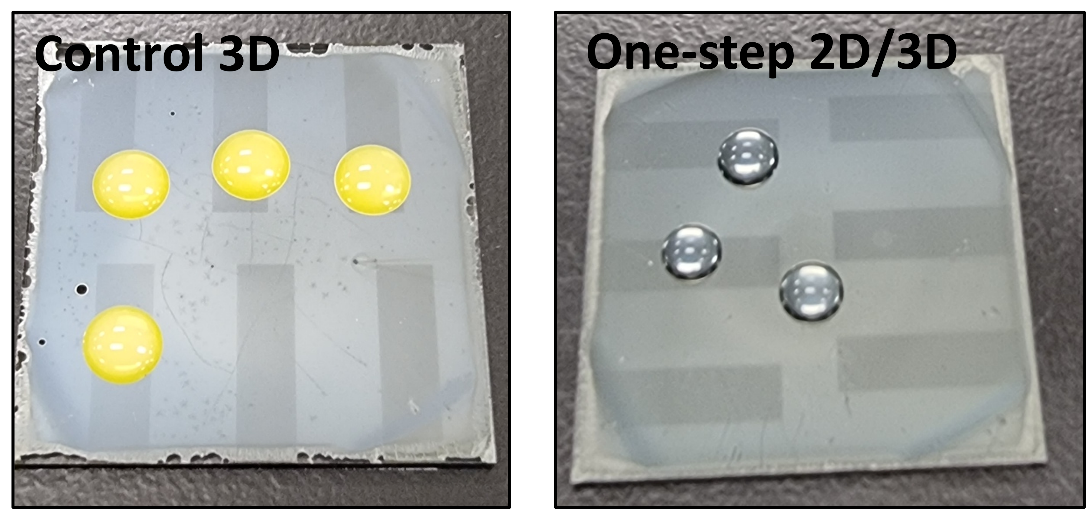


**Fig. S16** Water drop test of control 3D and 2D/3D treated film perovskite using oleylammonium iodide ligands


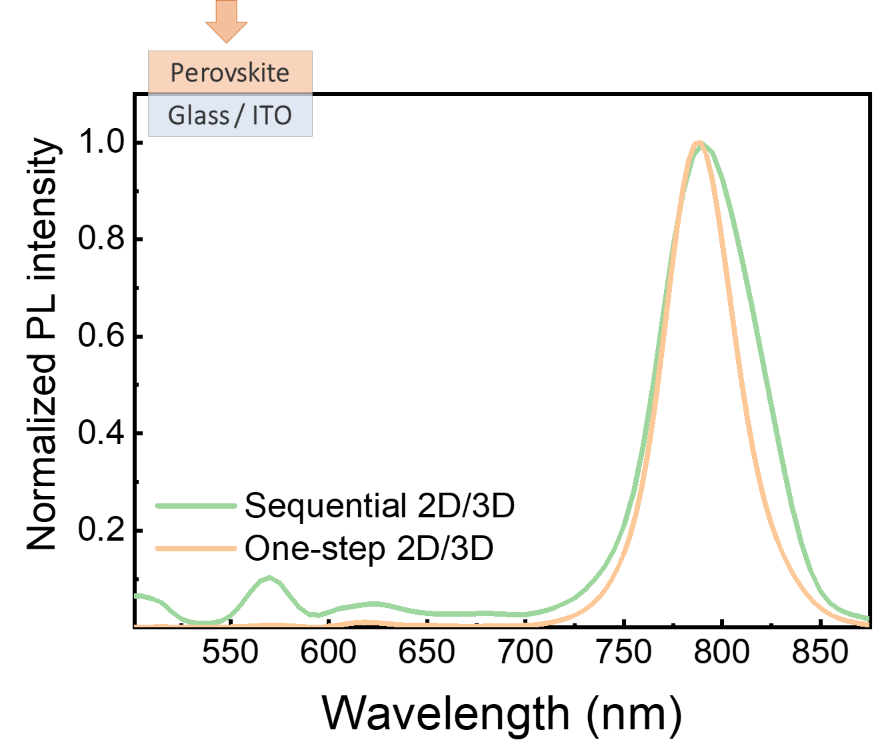


**Fig. S17** PL mapping of sequential 2D/3D and one-step 2D/3D from the top side on the ITO/Perovskite film


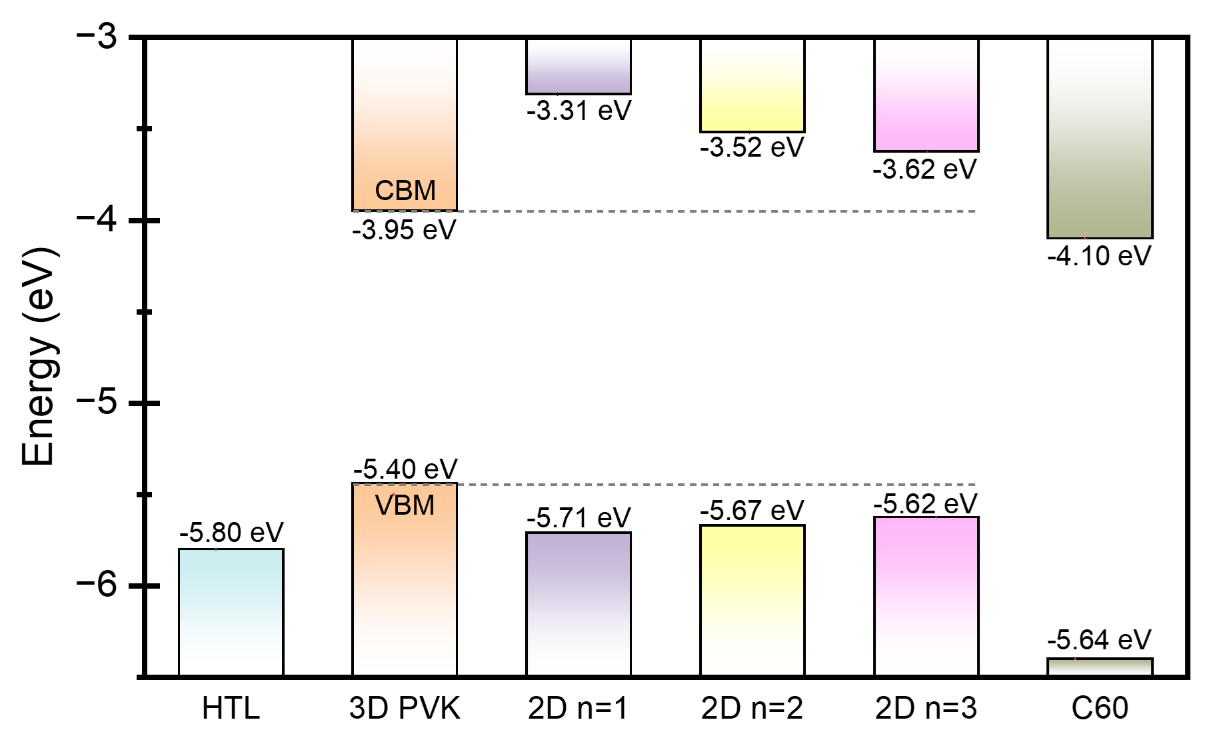


**Fig. S18** Illustration of energy band alignment of 2D/3D perovskite bilayer generated from PESA, PL and UPS


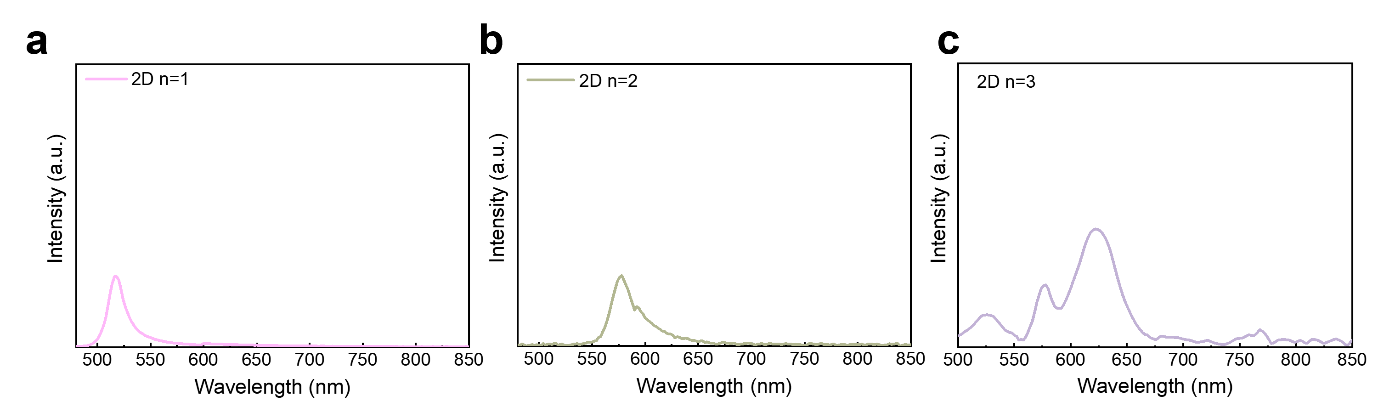


**Fig. S19** PL spectra of pure and near pure phase 2D perovskite film for (a) n=1 (b) n=2, and (c) n=3


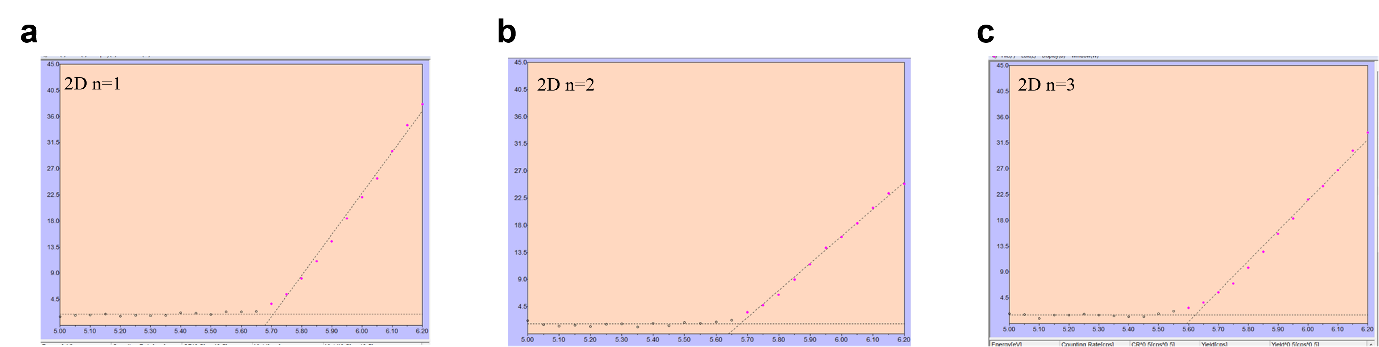


**Fig. S20** PESA of pure and near pure phase 2D perovskite film for (a) n=1 (b) n=2, and (c) n=3


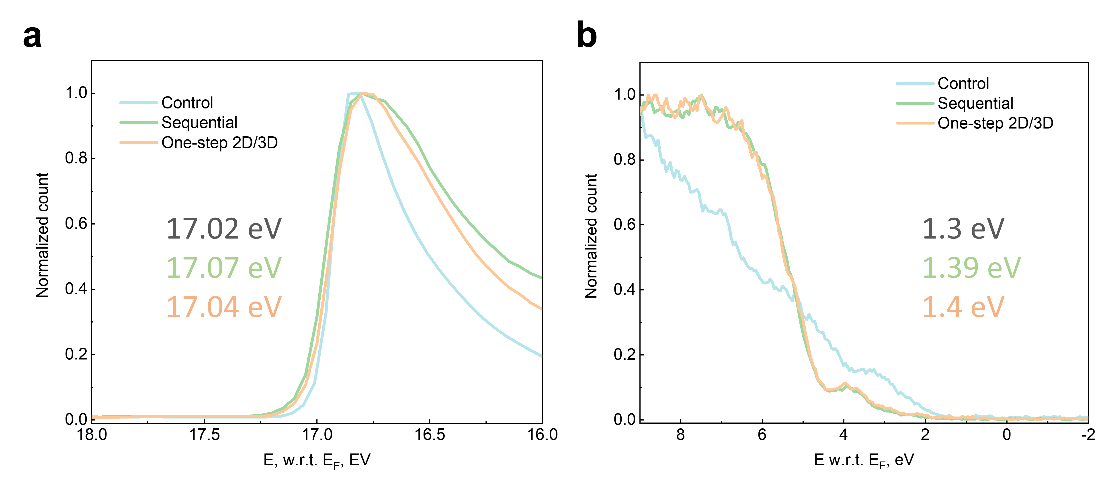


**Fig. S21** (**a-b**) Secondary electron cut-off and valence spectra plots from UPS measurement


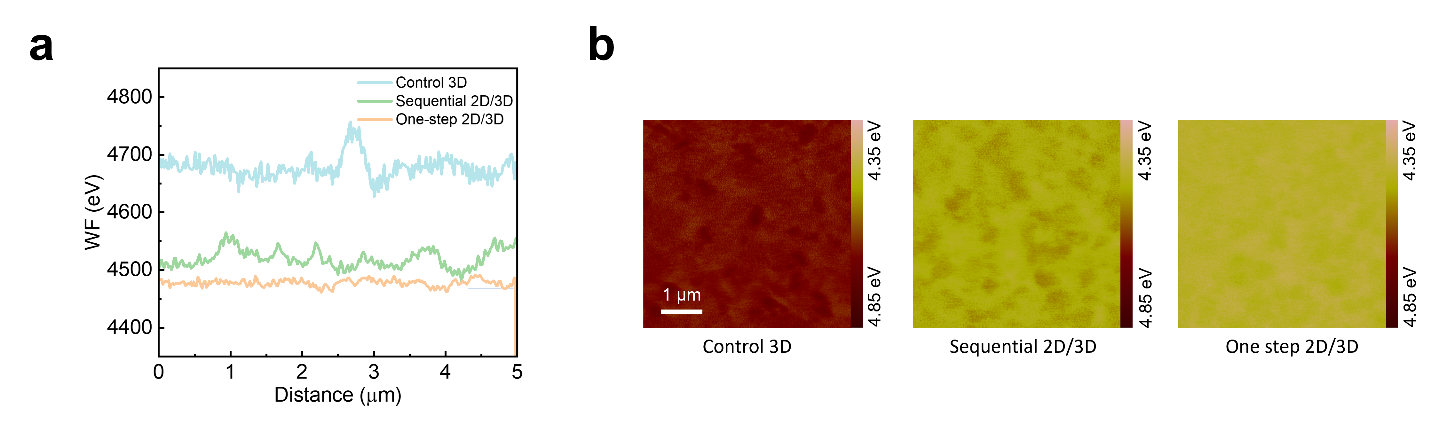


**Fig. S22** KPFM mapping of perovskite using of control 3D, sequential 2D/3D, and one-step 2D/3D


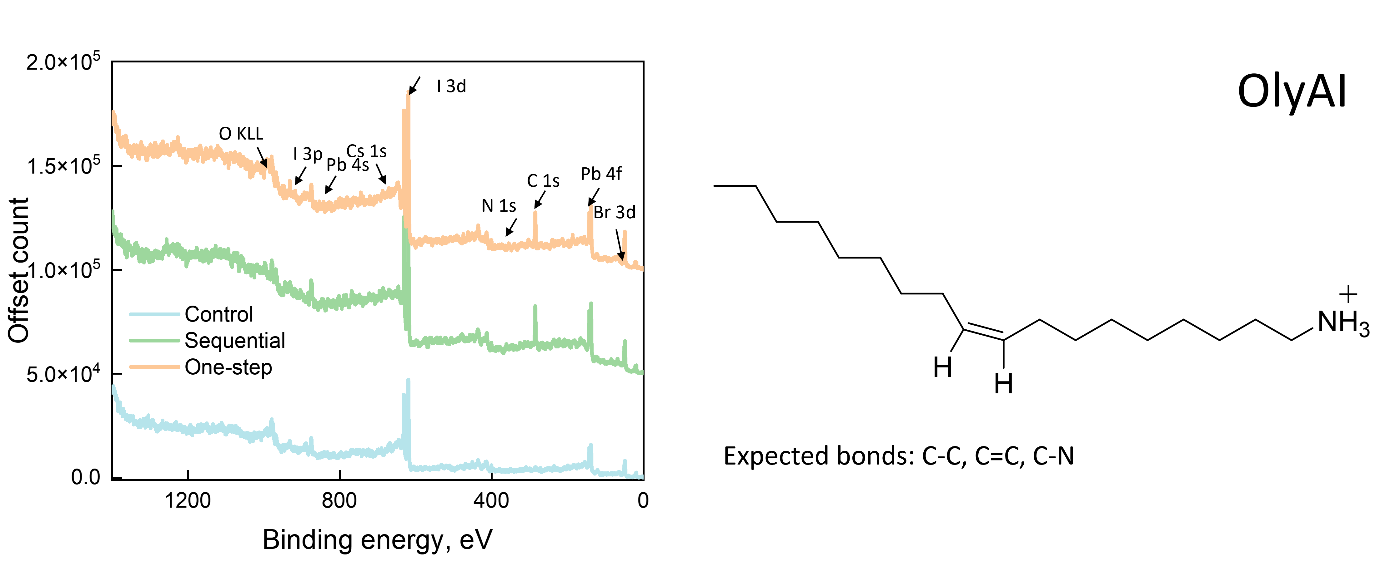


**Fig. S23** XPS Survey scan of the perovskite elements for control 3D, sequential 2D/3D, and one-step 2D/3D technique, showing no additional impurities to the system


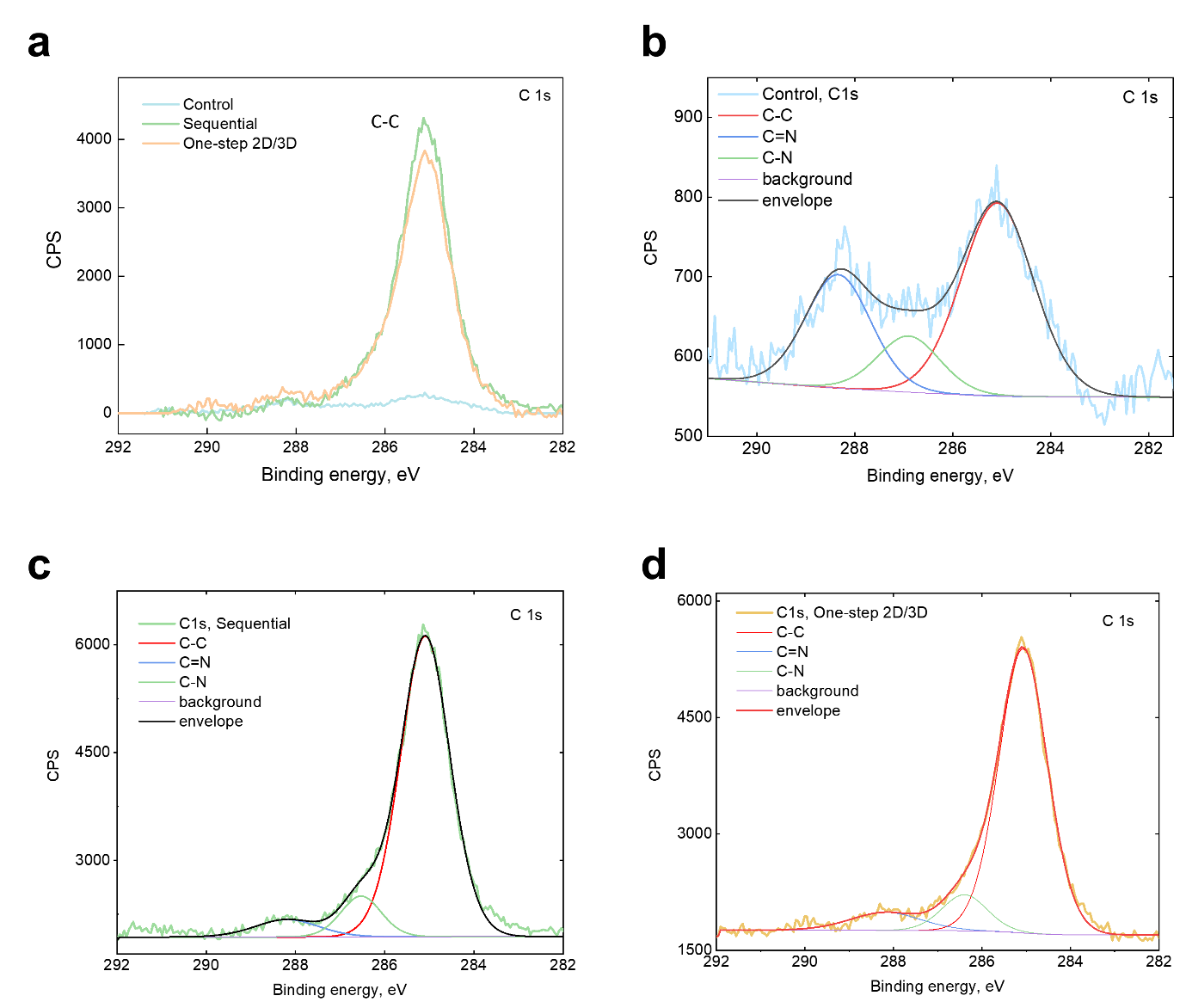


**Fig. S24** (**a-d**) High-resolution spectra of C1s region with deconvoluted peaks for various C bonding in control 3D, sequential 2D/3D, and one-step 2D/3D


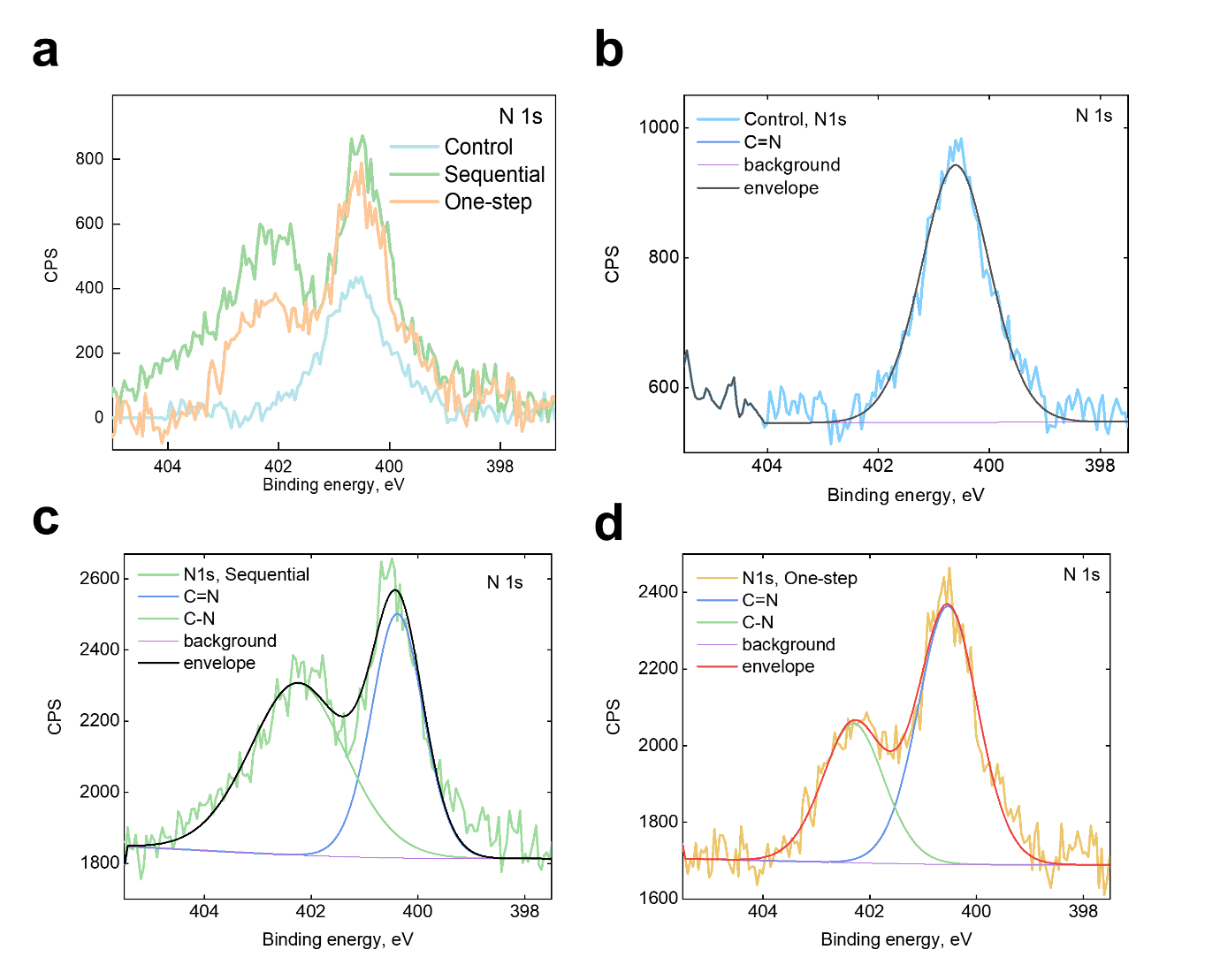


**Fig. S25** (**a-d**) High-resolution spectra of N1s region with deconvoluted peaks for various C bonding in control 3D, sequential 2D/3D, and one-step 2D/3D


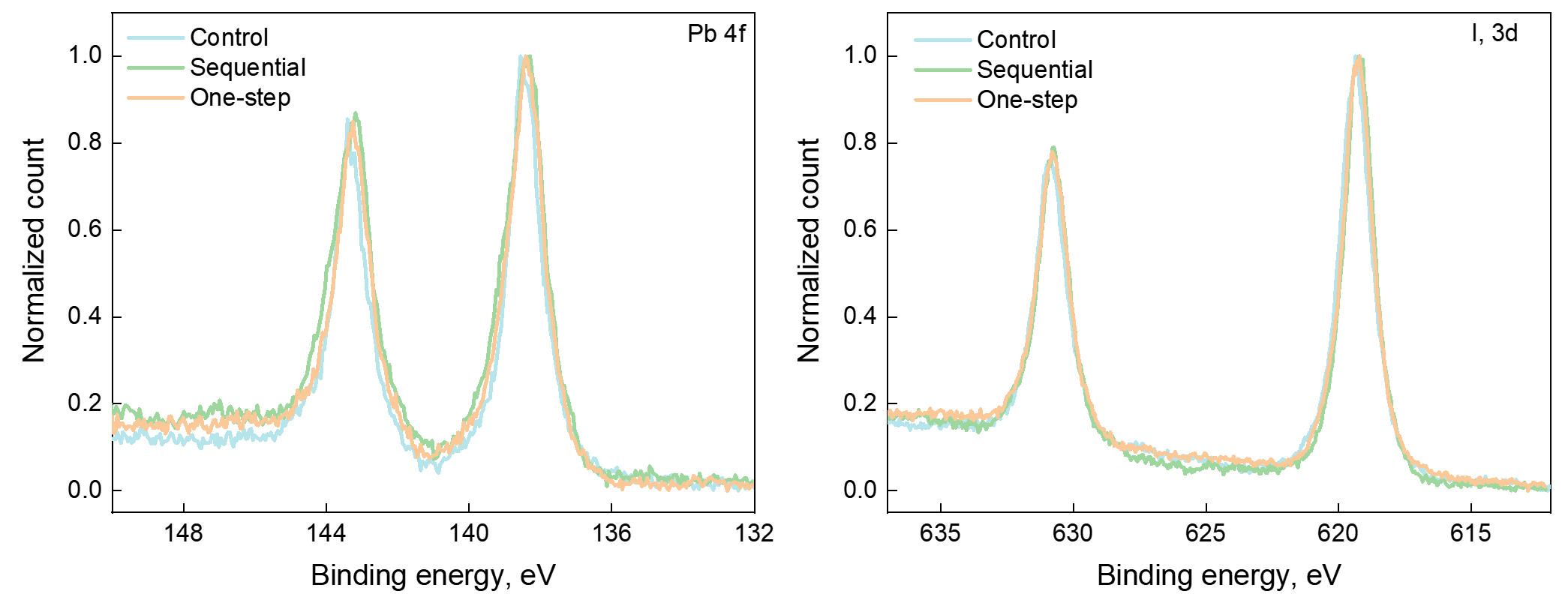


**Fig. S26** Pb 4f, I 3d peak from XPS analysis for control, sequential, and one-step technique perovskite film


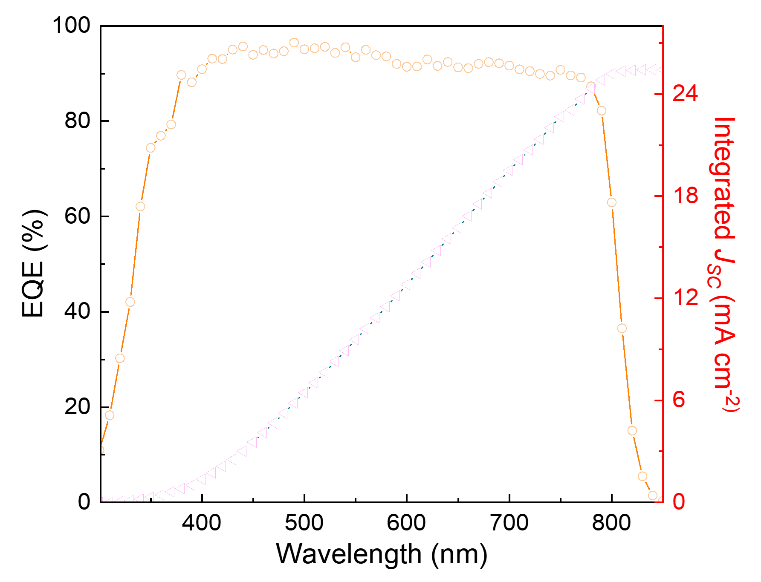


**Fig. S27** External quantum efficiency (EQE) plot of device prepared by one-step 2D/3D method using 1.55 eV perovskite with a composition of Cs_0.025_MA_0.075_FA_0.90_PbI_3_

**Table S1** Device performance parameters

|  | V_OC_ (V) | J_SC_ (mA/cm^2^) |  | FF | PCE % |
| --- | --- | --- | --- | --- | --- |
| Control 3D | 1.120 | 25.30 |  | 81.11 | 23.20 |
| Sequential 2D/3D | 1.170 | 25.40 |  | 81.11 | 24.30 |
| One-step 2D/3D | 1.198 | 25.89 |  | 85.12 | 26.22 |


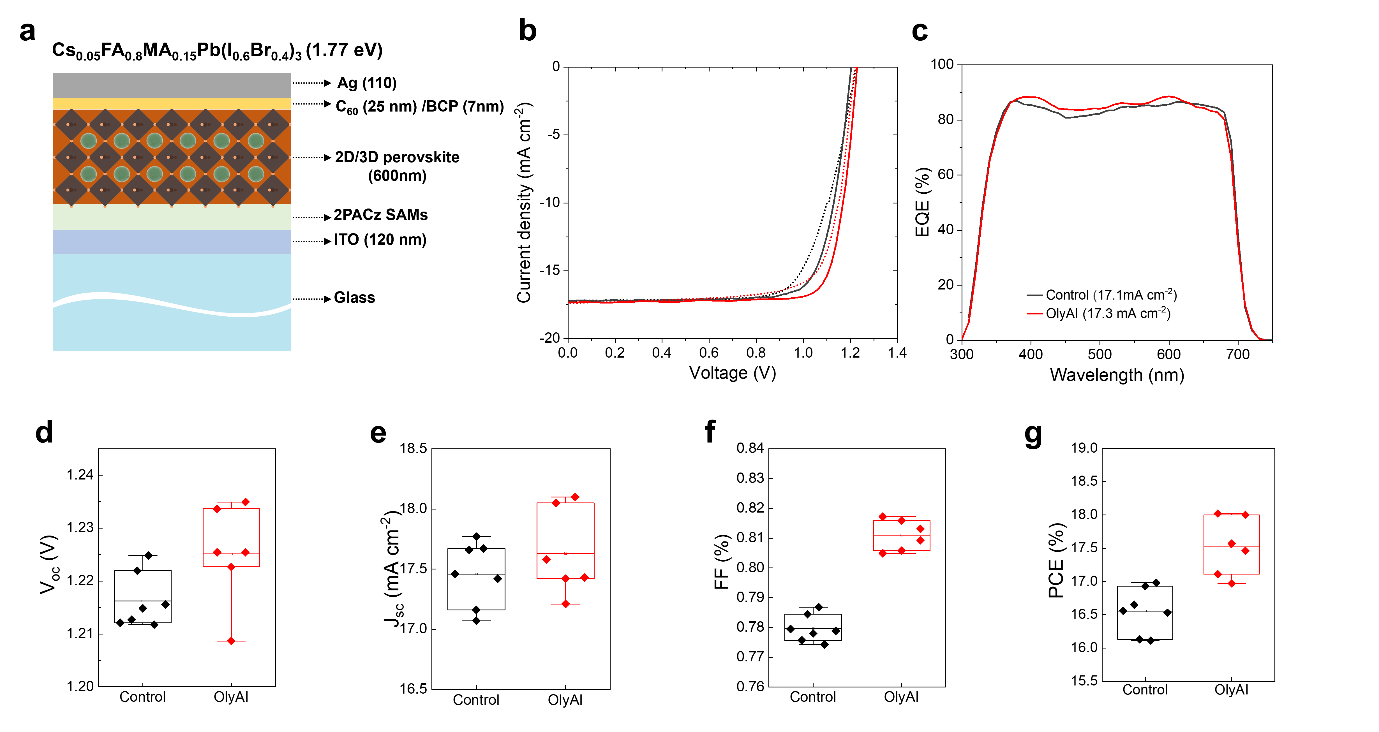


**Fig. S28** device performance and parameter under different band gap perovskite (1.77 eV) (**a**) device structure, (**b**) J-V curve, (**c**) EQE, (**d**) V_OC_, (**e**) J_SC_, (**f**) FF, and (**g**) PCE for device with control 3D and one-step 2D/3D


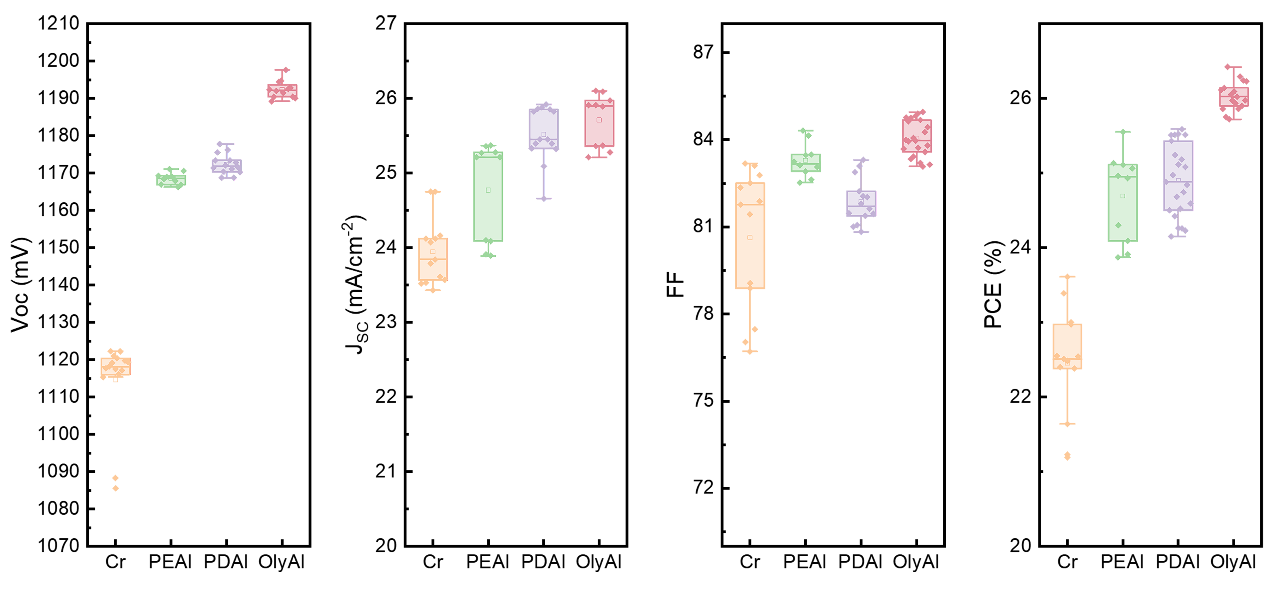


**Fig. S29** comparison of device performance using different ligands via one-step 2D/3D technique


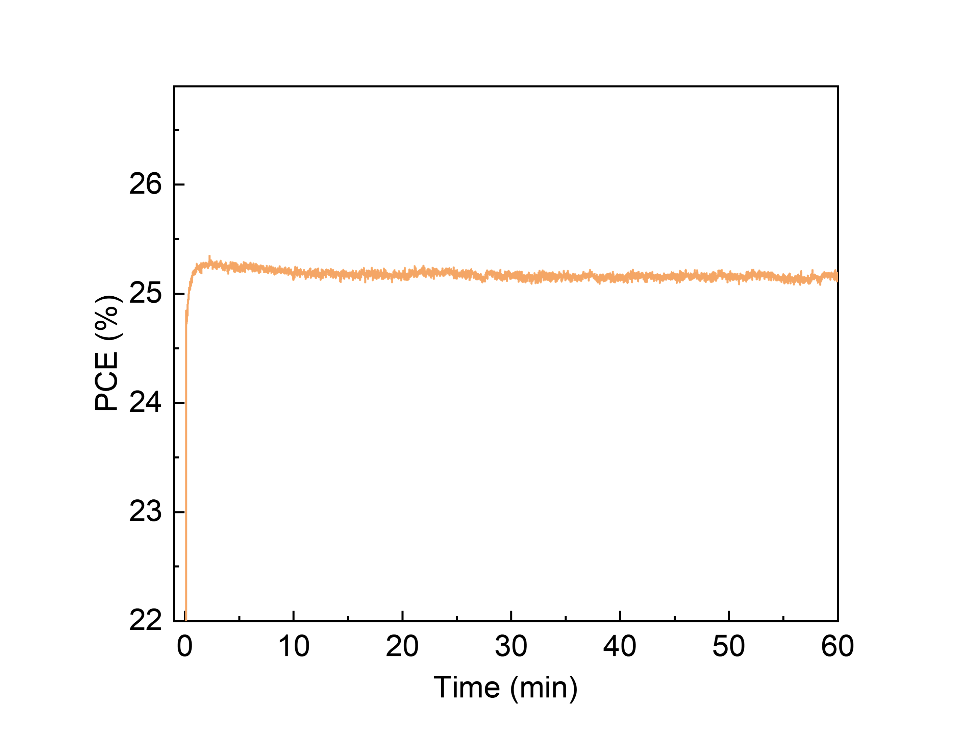


**Fig. S30** SPO of champion device prepared by one-step 2D/3D method


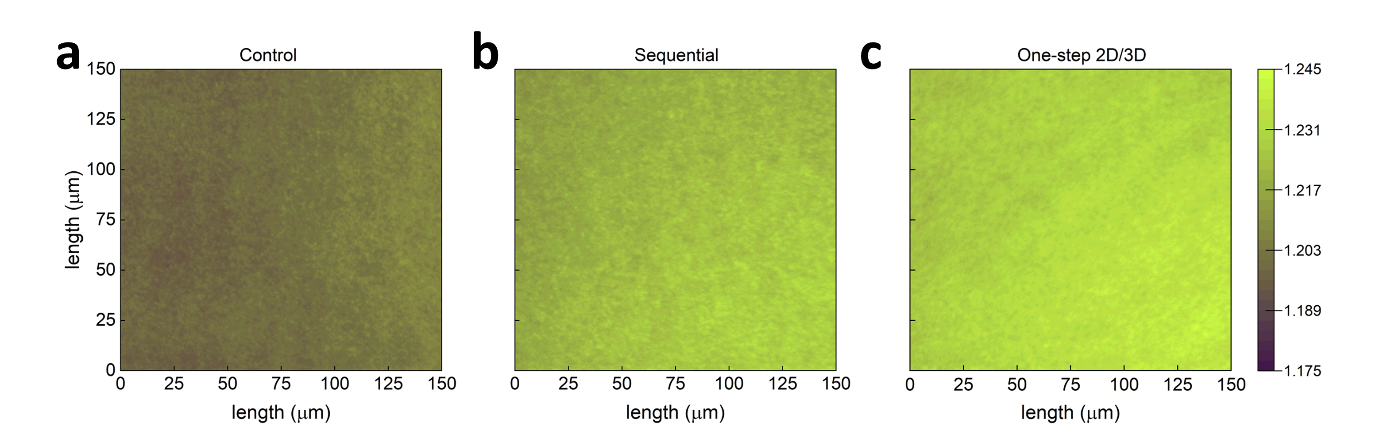


**Fig. S31** QFLS values extracted from absolute PL images for (**a**) control 3D, (**b**) sequential 2D, and (**c**) one-step 2D/3D perovskite films fabricated on glass/ITO substrates
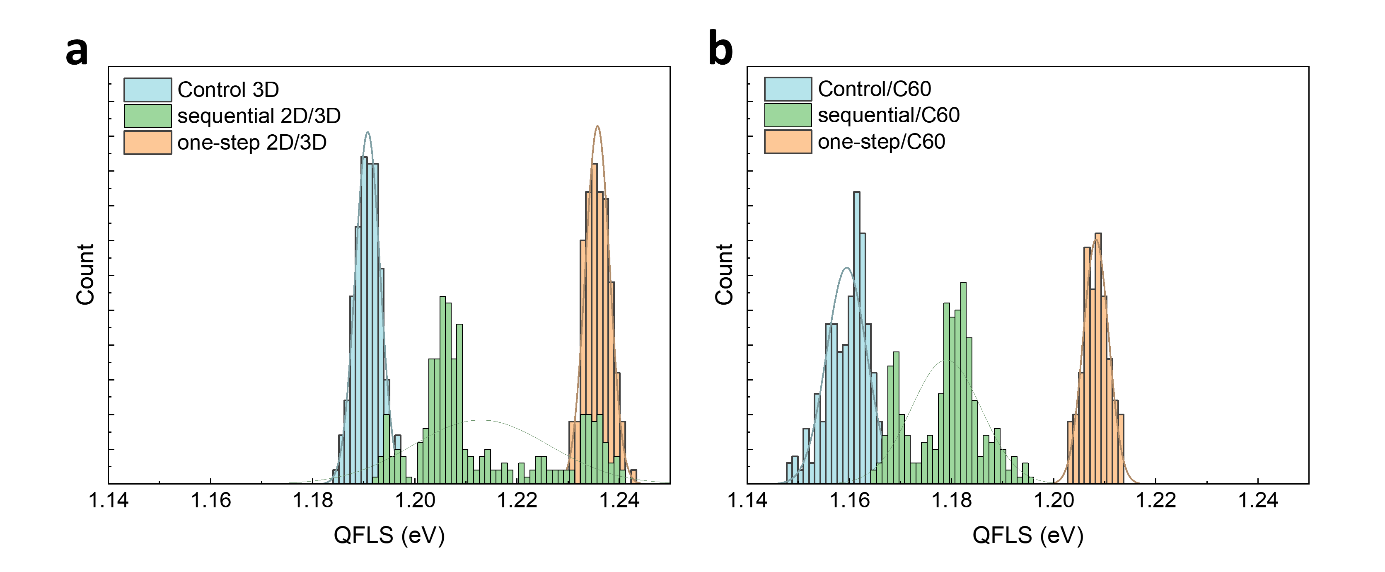
**Fig. S32** Histogram of QFLS values distribution for control 3D, sequential 2D/3D, and one-step 2D/3D perovskite films for (**a**) glass/ITO/perovskite and (**b**) glass/ITO/perovskite/C_60_ stacks


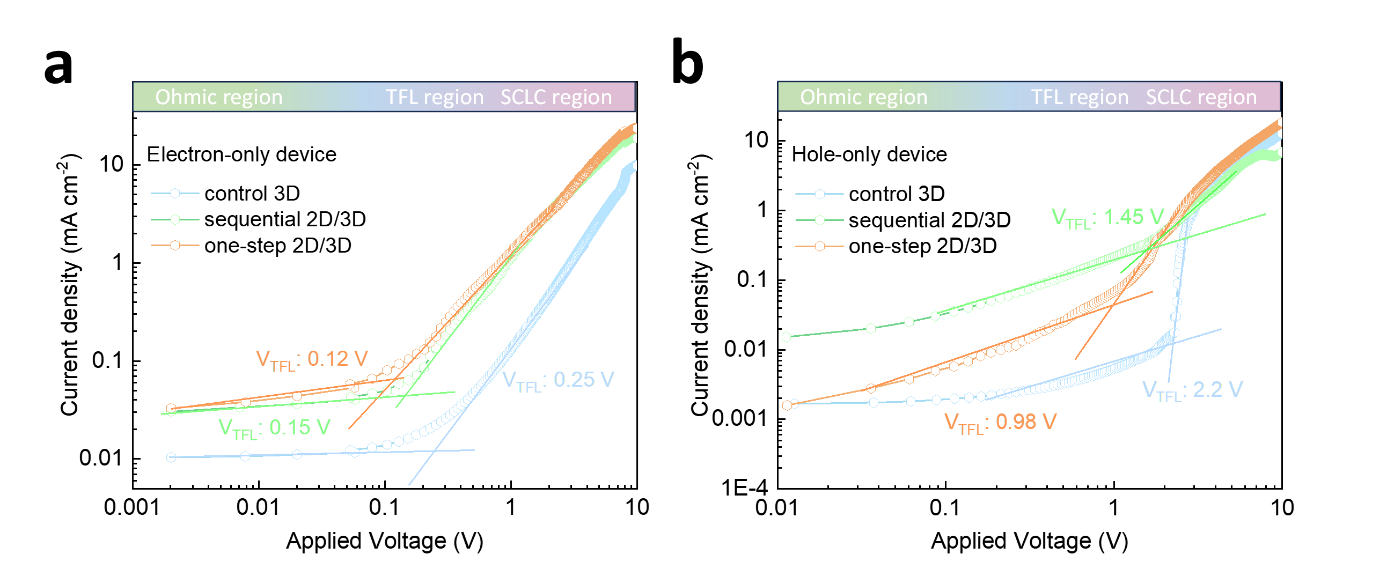


**Fig. S33** SCLC plots for (**a**) electron only, and (**b**) hole-only devices prepared by control 3D, sequential 2D/3D, and one-step 2D/3D methods

**
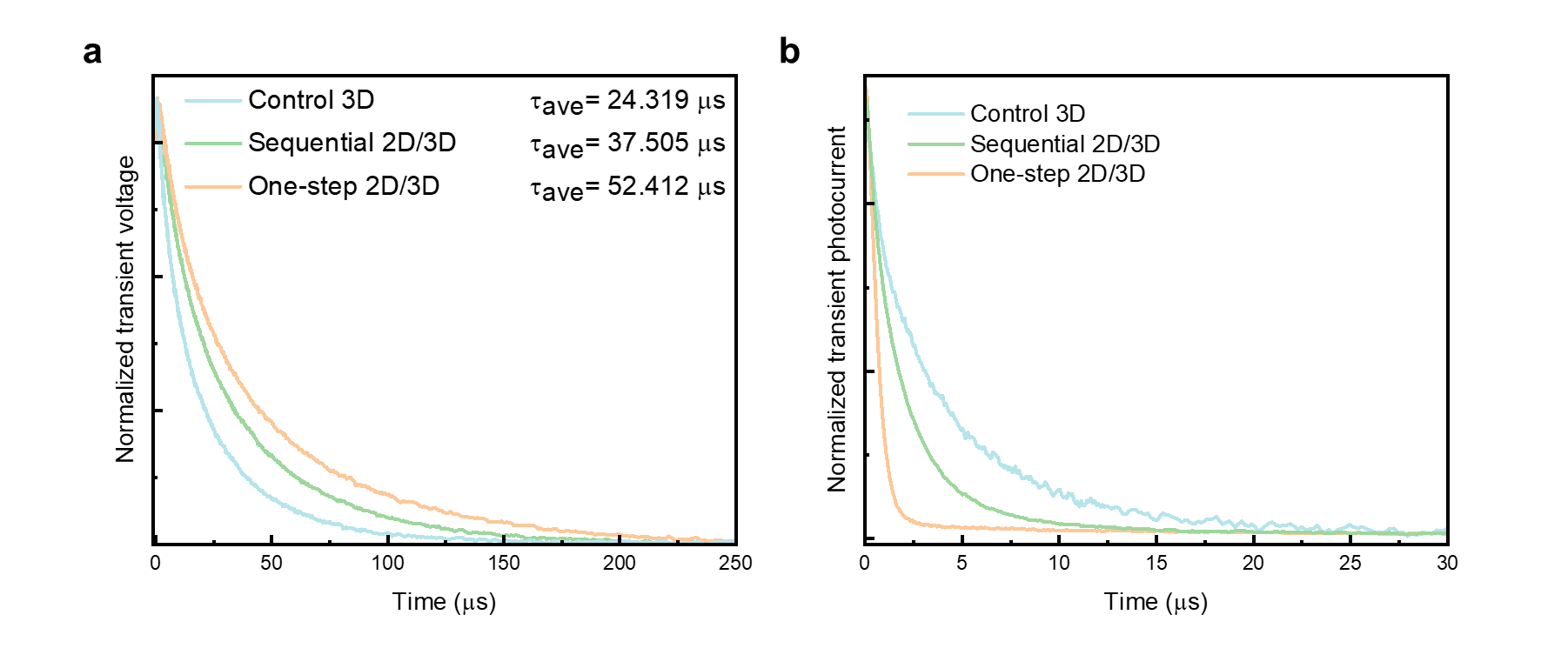
Fig. S34** (**a**) TPV measurement of control 3D, sequential 2D/3D, and one-step 2D/3D, and (**b**) TPC measurement of control 3D, sequential 2D/3D, and one-step 2D/3D


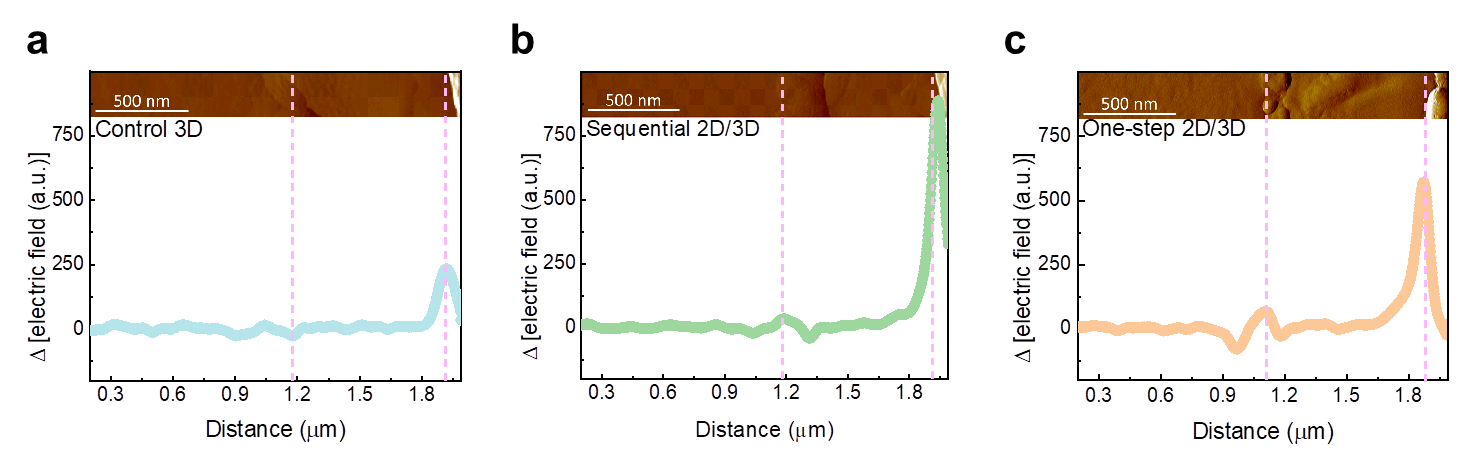


**Fig. S35** Electric field distribution through both interfaces from the C_60_ electron transport layer to the glass/ITO using cross-sectional KPFM measurements under positive voltage bias for (**a**) control 3D, (**b**) sequential 2D, and (**c**) one-step 2D/3D perovskites


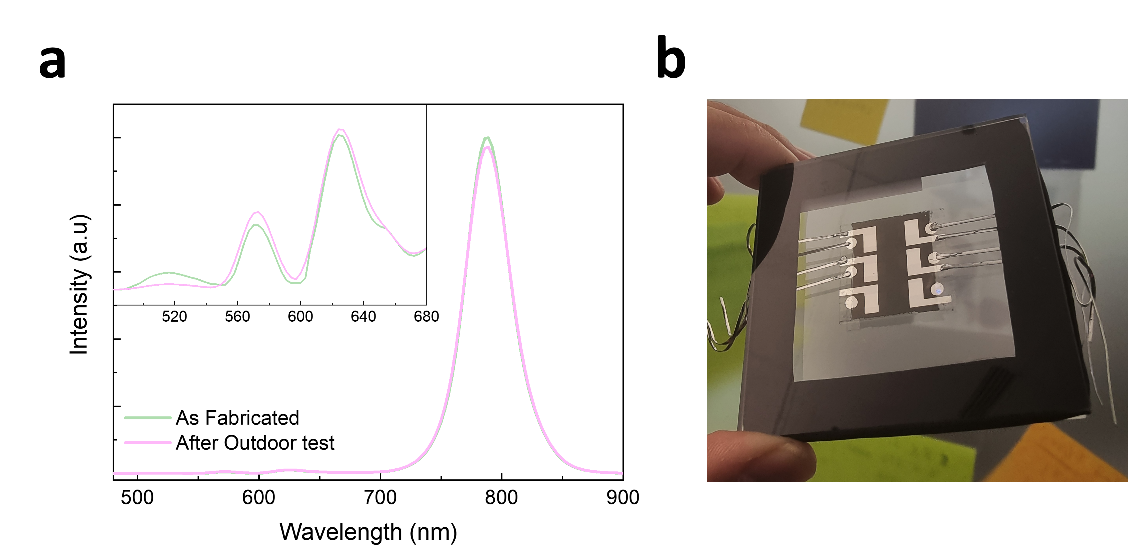
**Fig. S36** (**a**) photoluminescence peak from fresh fabricated perovskite and aged perovskite from our outdoor test after 1000 h (**b**) encapsulated sample for outdoor test


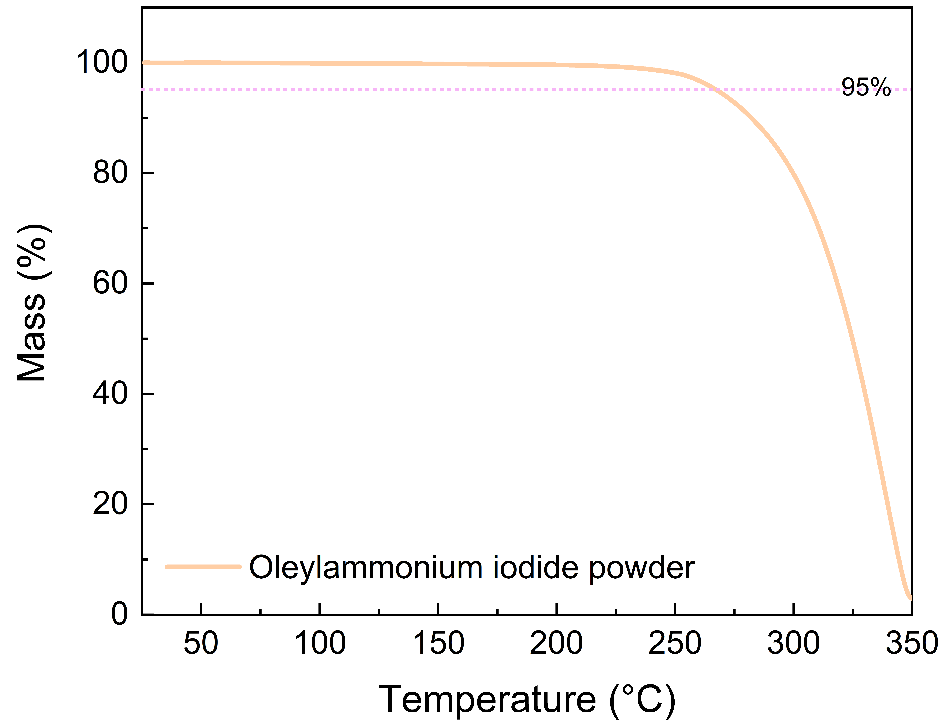


**Fig. S37** TGA analysis of OlyA^+^ ligand


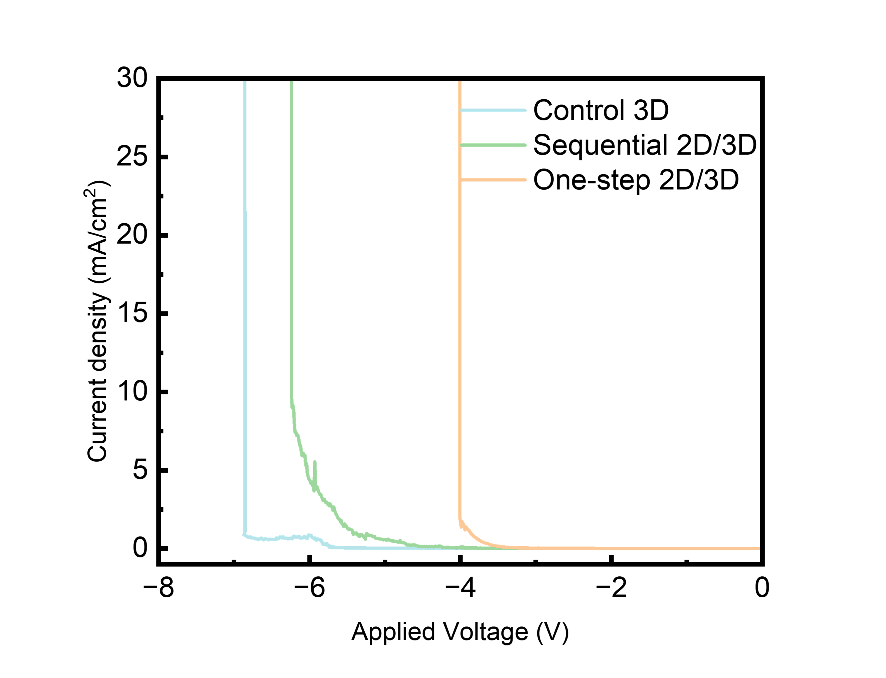


**Fig. S38** Reverse bias of control 3D, sequential 2D/3D, and one-step 2D/3D methods

**Supplementary References**

1. J. Liu, M. De Bastiani, E. Aydin, G.T. Harrison, Y. Gao et al., Efficient and stable perovskite-silicon tandem solar cells through contact displacement by MgF(x). Science **377**(6603), 302–306 (2022). <https://doi.org/10.1126/science.abn8910>
2. G. Kresse, D. Joubert, From ultrasoft pseudopotentials to the projector augmented-wave method. Phys. Rev. B **59**(3), 1758–1775 (1999). <https://doi.org/10.1103/physrevb.59.1758>
3. J.P. Perdew, K. Burke, M. Ernzerhof, Generalized gradient approximation made simple. Phys. Rev. Lett. **77**(18), 3865–3868 (1996). <https://doi.org/10.1103/physrevlett.77.3865>
4. S. Grimme, J. Antony, S. Ehrlich, H. Krieg, A consistent and accurate *ab initio* parametrization of density functional dispersion correction (DFT-D) for the 94 elements H-Pu. J. Chem. Phys. **132**(15), 154104 (2010). https://doi.org/10.1063/1.3382344
5. L. Bengtsson, Dipole correction for surface supercell calculations. Phys. Rev. B **59**(19), 12301–12304 (1999). <https://doi.org/10.1103/physrevb.59.12301>
6. V. Barone, M. Cossi, Quantum calculation of molecular energies and energy gradients in solution by a conductor solvent model. J. Phys. Chem. A **102**(11), 1995–2001 (1998). <https://doi.org/10.1021/jp9716997>
7. M. Cossi, N. Rega, G. Scalmani, V. Barone, Energies, structures, and electronic properties of molecules in solution with the C-PCM solvation model. J. Comput. Chem. **24**(6), 669–681 (2003). <https://doi.org/10.1002/jcc.10189>
8. M.J. Frisch, G.W. Trucks, H.B. Schlegel, G.E. Scuseria et al., Gaussian 16 rev. C.01. (2016).
